# Crystal structure and dynamics of a lipid-induced potential desensitized-state of a pentameric ligand-gated channel

**DOI:** 10.7554/eLife.23886

**Published:** 2017-03-06

**Authors:** Sandip Basak, Nicolaus Schmandt, Yvonne Gicheru, Sudha Chakrapani

**Affiliations:** 1 Department of Physiology and Biophysics, School of Medicine, Case Western Reserve University, Cleveland, United States; 2 Department of Neuroscience, School of Medicine, Case Western Reserve University, Cleveland, United States; University of Wisconsin-Madison, United States

**Keywords:** Cys-loop receptors, allosteric modulation, nanodiscs, site-directed spin labeling, *E. coli*

## Abstract

Desensitization in pentameric ligand-gated ion channels plays an important role in regulating neuronal excitability. Here, we show that docosahexaenoic acid (DHA), a key ω−3 polyunsaturated fatty acid in synaptic membranes, enhances the agonist-induced transition to the desensitized state in the prokaryotic channel GLIC. We determined a 3.25 Å crystal structure of the GLIC-DHA complex in a potentially desensitized conformation. The DHA molecule is bound at the channel-periphery near the M4 helix and exerts a long-range allosteric effect on the pore across domain-interfaces. In this previously unobserved conformation, the extracellular-half of the pore-lining M2 is splayed open, reminiscent of the open conformation, while the intracellular-half is constricted, leading to a loss of both water and permeant ions. These findings, in combination with spin-labeling/EPR spectroscopic measurements in reconstituted-membranes, provide novel mechanistic details of desensitization in pentameric channels.

**DOI:** http://dx.doi.org/10.7554/eLife.23886.001

## eLife digest

The nerve cells (or neurons) in the brain communicate with each other by releasing chemicals called neurotransmitters that bind to ion channels on neighboring neurons. This ultimately causes ions to flow in or out of the receiving neuron through these ion channels; this ion flow determines how the neuron responds.

One family of ion channels that is found at the junction between neurons, and between neurons and muscle fibers, is known as the pentameric ligand-gated ion channels (or pLGICs). These channels act as ‘gates’ that open to allow ions through them when a neurotransmitter binds to the channel. In addition to the open ‘active’ state, the channels can take on two different ‘inactive’ states that do not allow ions to pass through the channel: a closed (resting) state and a desensitized state (that is still bound to the neurotransmitter). Understanding how channels switch between these states is important for designing drugs that correct problems that cause the channels to work incorrectly.

Problems that affect the desensitized state have been linked to neurological disorders such as epilepsy. Medically important molecules such as anesthetics and alcohols are thought to affect desensitization, and drugs that target desensitized ion channels may present ways of treating neurological disorders with fewer side effects.

Docosahexaenoic acid (DHA) is an abundant lipid molecule that is present in the membranes of neurons. It is one of the key ingredients in fish oil supplements and is thought to enhance learning and memory. DHA affects the desensitization of pLGICs but it is not clear exactly how it does so.

Basak et al. now show that DHA affects a bacterial pLGIC in the same way as it affects human channels – by enhancing desensitization. Using a technique called X-ray crystallography to analyze the channel while bound to DHA revealed a previously unobserved channel structure. The DHA molecule binds to a site at the edge of the channel and causes a change in its structure that leaves the upper part of the channel open while the lower part is constricted. Basak et al. predict that molecules such as anesthetics target this desensitized state.

The next step will be to obtain the structures of bacterial and human pLGIC channels in a natural membrane environment. This will allow us to better understand the changes in structure that the channels go through as they transmit signals between neurons, and so help in the development of new treatments for neurological disorders.

**DOI:** http://dx.doi.org/10.7554/eLife.23886.002

## Introduction

**Figure 1.**
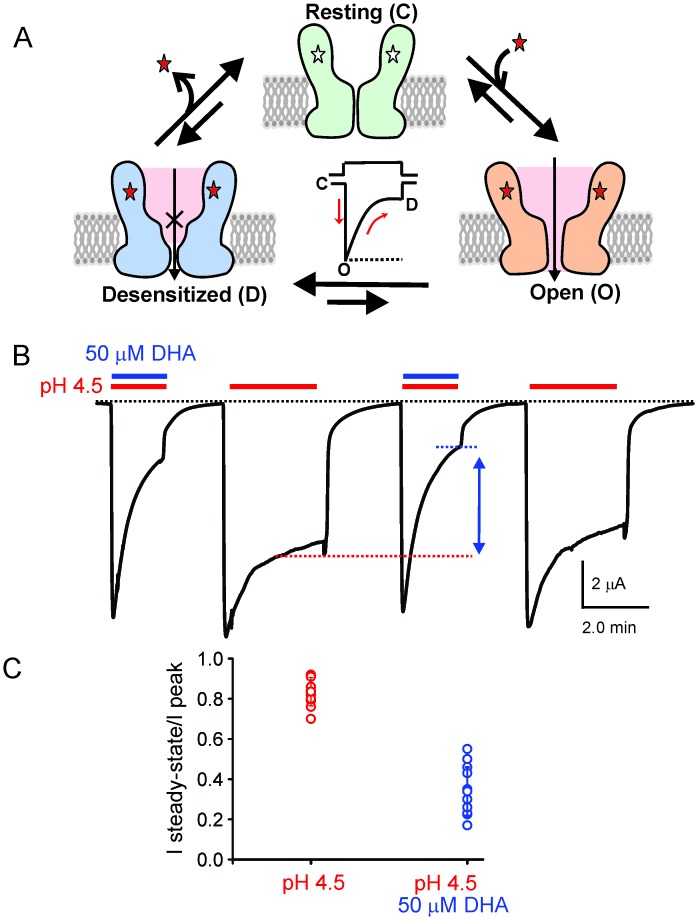
DHA modulation of GLIC function. (**A**) A minimal gating scheme showing three fundamental conformational states that constitute pLGIC function: a resting state [C], a transient open-state [O], and a desensitized state [D]. Agonist-binding shifts the equilibrium towards the high-affinity D state such that under steady-state conditions, the channels are predominantly in the desensitized state. Allosteric modulators exert their effect by altering the transition, and hence the equilibrium, between the three states. (**B**) The trace shows a continuous recording of GLIC currents in oocytes measured by two electrode voltage-clamp (TEVC) in response to multiple pH-4.5 pulses. The pH-pulses were interspaced by perfusion with the pH 7.4 solution (for deactivation and recovery). Currents were measured in the absence (*marked by red lines*) or presence of 50 μM DHA (*marked by red and blue lines*). The baseline is marked as a dotted black line. DHA inhibits GLIC currents by increasing desensitization (faster current decay and lower steady-state currents; highlighted by the vertical blue arrow and dotted blue/red lines). The effect of DHA on the current decay was fully reversible, as seen in the second and fourth pH-pulses. (**C**) A plot of the ratio of steady-state (measured at t = 2.2 min) over the peak current amplitude for the two conditions (n = 12) with s.d shown as error bars. **DOI:** http://dx.doi.org/10.7554/eLife.23886.003

Fast synaptic transmission throughout the central and peripheral nervous system is mediated by pentameric ligand-gated ion channels (pLGICs), also referred to as Cys-loop receptors. The vertebrate channels of this superfamily include both inhibitory anion-selective channels (γ-aminobutyric acid receptors- GABA_A_R and glycine receptors- GlyR) and excitatory cation-selective channels (nicotinic acetylcholine receptors- nAChR and serotonin receptors- 5HT_3A_R). The binding of a neurotransmitter initiates a cascade of protein motions that lead to transitions between resting, open, and desensitized conformations (Unwin and Fujiyoshi, 2012; Du et al., 2015; Sauguet et al., 2014; Althoff et al., 2014) (Figure 1A). Ionic fluxes, and hence the post-synaptic responses, are critically governed by the transition rates and equilibrium populations of these functional states. Aberration of these molecular events underlies many neurological disorders and therefore, pLGICs are therapeutic targets for treating these conditions. To develop a molecular understanding of the pLGIC gating mechanism and its modulation requires high-resolution structures of the channel in multiple functional states. While there has been ground-breaking progress in determining the structures of several members of the pLGIC family, from both prokaryotic and eukaryotic origin, an unequivocal assignment of functional states to these conformations has not been achieved (Unwin and Fujiyoshi, 2012; Du et al., 2015; Hilf and Dutzler, 2009, 2008; Sauguet et al., 2013; Bocquet et al., 2009; Miller and Aricescu, 2014; Hassaine et al., 2014; Hibbs and Gouaux, 2011). Structural mechanisms underlying channel opening (Du et al., 2015; Althoff et al., 2014; Sauguet et al., 2013; Velisetty et al., 2012) and desensitization have been areas of extensive investigation (Du et al., 2015; Miller and Aricescu, 2014; Morales-Perez et al., 2016).

**Figure 1—figure supplement 1.**
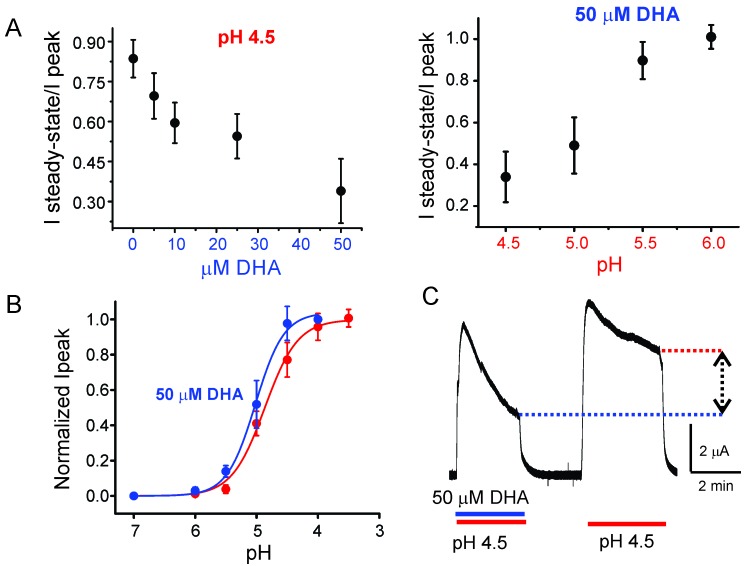
Effect of DHA on GLIC desensitization. (**A**) The effect of DHA at various concentrations (*left*) on currents at pH 4.5 and the effect of 50 μM DHA on currents elicited by various extracellular pH (*right*). The currents were recorded by TEVC at a holding potential of −60 mV. In each case, a ratio of the steady-state current (measured at t = 4.5 min) to the peak amplitude was plotted. The error bars denote s.d (n = 6) (**B**) Normalized peak amplitudes in the presence (blue) and absence (red) of 50 μM DHA plotted as a function of pH, and the data were fitted with the Hill equation to yield pH_50_4.87 ± 0.04 and n_H_1.6 ± 0.2 in the absence of DHA; pH_50_5.02 ± 0.03 and n_H_1.9 ± 0.3 in the presence of DHA. The error bars denote s.d (n = 3) (**C**) Outward currents recorded at pH 4.5 and +60 mV holding potential in the presence and absence of 50 μM DHA. The dashed lines and arrow mark the level of steady-state currents under the two conditions. **DOI:** http://dx.doi.org/10.7554/eLife.23886.004

**Figure 1—figure supplement 2.**
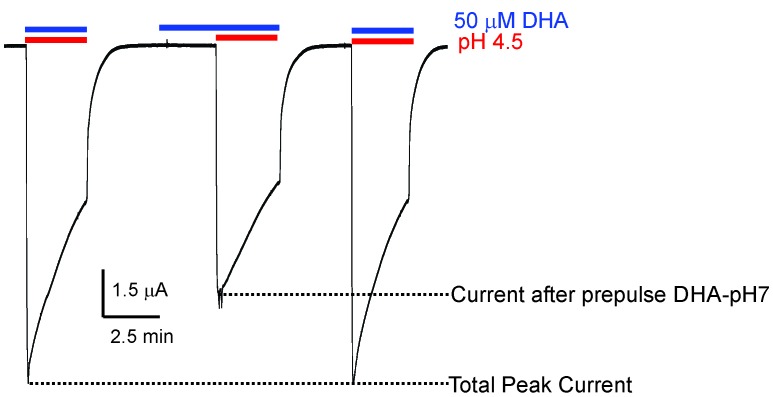
Effect of DHA-pre-application on GLIC currents. Representative pH-elicited GLIC-currents recorded by TEVC at −60 mV membrane potential in response to pre-application of 50 μM DHA at pH 7.4 (2.2 min duration) (middle trace) and compared with currents recorded without DHA pre-application (first and third pulses). All the three pH 4.5-pulses had 50 μM DHA. The ratio of the peak-current amplitudes measured with and without pre-application of DHA was 0.64 ± 0.11 (n = 5). **DOI:** http://dx.doi.org/10.7554/eLife.23886.005

Desensitization regulates the frequency and amplitude of synaptic response during fast, repetitive stimulation and is implicated to play a role in the synaptic plasticity of neural networks associated with learning, memory, and attention (Giniatullin et al., 2005; Elenes et al., 2006; Jones and Westbrook, 1996). Mutations in nAChR, GABA_A_R, and GlyR that lead to altered desensitization kinetics have been associated with congenital myasthenic syndrome, frontal lobe epilepsy, and human startle disease (Bertrand et al., 2002; Matsushima et al., 2002; De Fusco et al., 2000; Sine et al., 2002; Saul et al., 1999; Bowser et al., 2002). Desensitization is modulated by a range of endogenous and exogenous factors, including Ca^2+^, membrane lipids (cholesterol, anionic lipids, and polyunsaturated fatty acids), neurosteroids, alcohols, and anesthetics (Arias, 1998; Fenster et al., 1999; Huganir and Greengard, 1990). In particular, positive allosteric modulators of nAChR, which slow desensitization, are being developed for the treatment of Alzheimer’s disease, schizophrenia, depression, and pain (Gopalakrishnan et al., 2007; Donnelly-Roberts et al., 2011; Hurst et al., 2005; Young et al., 2008). Strategies targeting desensitization and allosteric modulation are being considered as a means to design safer therapeutics that have fewer side effects (Buccafusco et al., 2009).

It is now well established that membrane-lipid constituents modulate gating transitions in many pLGIC members (Borroni et al., 2016; Baenziger and Corringer, 2011; Rankin et al., 1997; Sunshine and McNamee, 1992; Heidmann et al., 1980). In addition, they regulate the allosteric effects of alcohols, anesthetics, neurosteroids, and free fatty acids. These molecules partition into the lipid bilayer and affect channel properties by either interacting directly with the channel or by altering the interaction of the channel with surrounding lipids (Howard et al., 2014; Changeux, 2012; Campagna et al., 2003; Mihic et al., 1997). In particular, docosahexaenoic acid (DHA, an *ω*−3 polyunsaturated fatty acid with 22 carbons and six double bonds) has previously been reported to modulate nAChR and GABA_A_R function by increasing the rate and extent of desensitization (Hamano et al., 1996; Nabekura et al., 1998; Bouzat and Barrantes, 1993; Witt et al., 1999). DHA is a major polyunsaturated fatty acid (PUFA) in the brain and is found in high concentrations (up to 50 mol% of the total acyl chains of phospholipids) in synaptic plasma membranes. Reduced levels are linked with impaired learning ability (Hashimoto et al., 2016; Janssen and Kiliaan, 2014). DHA is readily esterified and incorporated into membrane phospholipids, altering both the physical properties of the membrane and the expression and function of associated membrane proteins. Free DHA released by intracellular enzymes such as phospholipase A_2_ and diacylglycerol lipase (Piomelli and Greengard, 1990) also influences membrane protein function (Moreno et al., 2012; Boland and Drzewiecki, 2008). While there are numerous reports describing the effects of DHA on several channel types, the mechanism of its action remains elusive (Bruno et al., 2007). Mutational analysis and subunit/isoform specific effects in a number of channels have implicated direct interaction with DHA (Ottosson et al., 2014; Hoshi et al., 2013a, 2013b), however evidence for a DHA binding site on these channels is still lacking.

Although the prokaryotic pLGIC are significantly less stringent in their need for specific membrane constituents, their function is modulated by lipids in an analogous way (Labriola et al., 2013; Velisetty and Chakrapani, 2012). Here, we show in electrophysiological recordings that desensitization in GLIC, a prokaryotic pH-gated pLGIC, is enhanced in the presence of DHA. GLIC has previously been crystallized in its resting (closed) and putative open states (Sauguet et al., 2014; Hilf and Dutzler, 2009; Bocquet et al., 2009), however, the desensitized state of the channel has been structurally elusive. Co-crystals of GLIC in the presence of DHA allowed us to successfully stabilize the channel in a novel state that is likely to be a desensitized conformation. The structure reveals a DHA molecule bound at the channel periphery, close to the M4 segment, and interacting with Arg118 in the Cys-loop (β6-β7 loop) through a salt-bridge. Both the M4 segment (also referred to as the ‘lipid-sensor’ in nAChR) and the Cys-loop are implicated in transducing agonist-induced conformational changes from across the extracellular domain (ECD) to the transmembrane domain (TMD). Mutations in the ECD-TMD interfacial region have been shown to affect desensitization (Bouzat et al., 2008). The most striking feature of our structure is the new pore conformation, which provides a molecular view of how ion permeation could potentially be occluded in a desensitized state.

## Results

### DHA modulates the pH-elicited response in GLIC

To test the effect of DHA on GLIC function, we expressed GLIC in *Xenopus laevis* oocytes and measured currents by two-electrode voltage-clamp (TEVC) techniques (Materials and methods). As previously shown, GLIC is activated by extracellular protons (pH 4.5) (Hilf and Dutzler, 2009; Bocquet et al., 2007) and the currents display a slow decay as the channels desensitize (Figure 1B). When DHA (50 μM) was co-applied with pH 4.5, the macroscopic decay from the peak was accelerated, leading to much smaller steady-state currents (Figure 1B, *blue arrow*). Upon deactivation at pH 7.0, subsequent pH change to 4.5 resulted in currents with peak amplitudes and decay phases indistinguishable from the first pulse, revealing that the effect of DHA was fully reversible. In addition to the effect on current decay, DHA decreases the amount of steady-state current (measured at 2.2 min from the start of application) as shown in the plot of the steady-state-to-peak ratio, suggesting that both the rate and the extent of desensitization are increased (Figure 1C). The Figure 1—figure supplement 1A shows a detailed analysis of the effect of DHA at different concentrations and at various activating pH. The effect on desensitization was observed at DHA concentrations above 5 μM, and was more pronounced at higher proton concentrations, suggesting that channel activation promotes the effect of DHA. Additionally, in the presence of DHA, a small left-shift in pH-response is observed for GLIC (Figure 1—figure supplement 1B). These findings are in fact expected for a modulator that promotes desensitization (the conformational state with the highest agonist-affinity). Further, outward current decay was also accelerated in the presence of DHA (Figure 1—figure supplement 1C), similar to the effect on inward currents. Upon pre-application of DHA (at pH 7.4) prior to co-application at pH 4.5, additional decrease in peak amplitudes is observed, suggesting that enhanced availability of DHA could result in larger effects on GLIC currents (Figure 1—figure supplement 2). The effects of DHA were fully reversible in all of the measured conditions.

**Figure 2.**
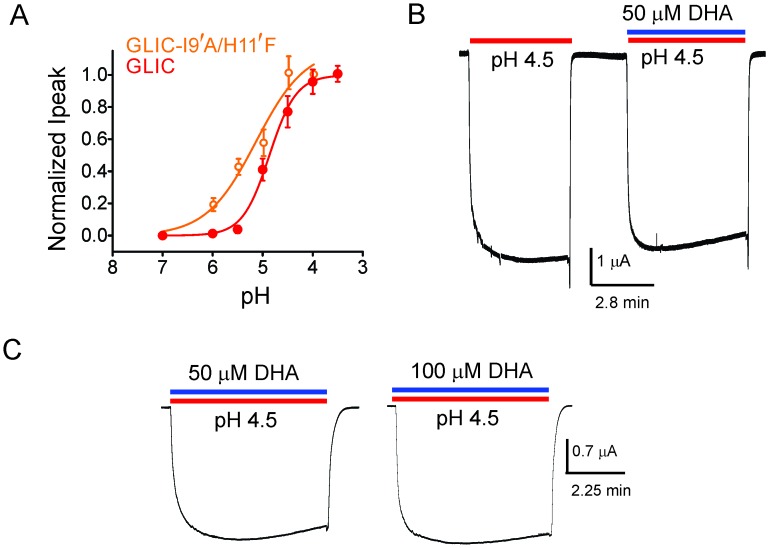
DHA has no effect on the non-desensitizing GLIC I9′A/H11′F mutant. (**A**) Normalized pH-response for GLIC-wt (red) and GLIC I9′A/H11′F double mutant (orange) at −60 mV. The error bars denote s.d and the curve is a fit to the Hill equation. GLIC-wt (pH_50_ 4.87 ± 0.04 and n_H_ 1.6 ± 0.2; n = 3) and GLIC I9′A/H11′F (pH_50_ 5.17 ± 0.19 and n_H_ 0.88 ± 0.29; n = 7). (**B**) Macroscopic currents measured by TEVC for GLIC I9′A/H11′F in response to pH jumps (from 7.4 to 4.5), at −60 mV holding potential, in the presence or absence of 50 μM DHA. (**C**) Currents recorded at pH 4.5 in the presence of either 50 μM DHA or 100 μM DHA. **DOI:** http://dx.doi.org/10.7554/eLife.23886.006

To further confirm that DHA indeed promotes an agonist-induced desensitized state rather than a pre-open resting state, we studied the effect of DHA on an alanine mutation at the Ile9’ position in M2. Mutation at the equivalent position in several pLGIC has been shown to increase agonist sensitivity and slow desensitization (Bocquet et al., 2007; Labarca et al., 1995; Filatov and White, 1995; Akabas et al., 1992; Yakel et al., 1993; Chang et al., 1996; Revah et al., 1991). The prediction is that perturbations that destabilize the desensitized state should lower the effect of DHA. The I9′A mutant exhibits a gain-of-function phenotype (Bocquet et al., 2007; Parikh et al., 2011; Gonzalez-Gutierrez et al., 2013) resulting in leaky oocytes, an effect that can be offset with a background mutation (H11′F [Wang et al., 2012; Rienzo et al., 2014]) that reduces pH-sensitivity (Schmandt et al., 2015). We found that the double-mutant (I9′A/H11′F) shows robust non-desensitizing currents with a pH_50_ 5.17 ± 0.19 (Figure 2A). Quite remarkably, DHA had no effect on this mutant over a range of pH conditions, and even up to a 100 μM concentration (Figure 2B and C, and Figure 2—figure supplement 1). Since the mutant’s pH response is close to wt, the DHA effect cannot be explained by stabilization of the resting or pre-open states. We would like to point out that the technical limitations of TEVC, which include slower perfusion rates, preclude us from resolving fast kinetic components of desensitization. We therefore, at this point, cannot ascertain which of the multiple desensitized states that DHA stabilizes. Nevertheless, above findings demonstrate that transitions to the desensitized state are necessary for the DHA effect, and that DHA may stabilize a desensitized conformation induced by the agonist during gating.

**Figure 2—figure supplement 1.**
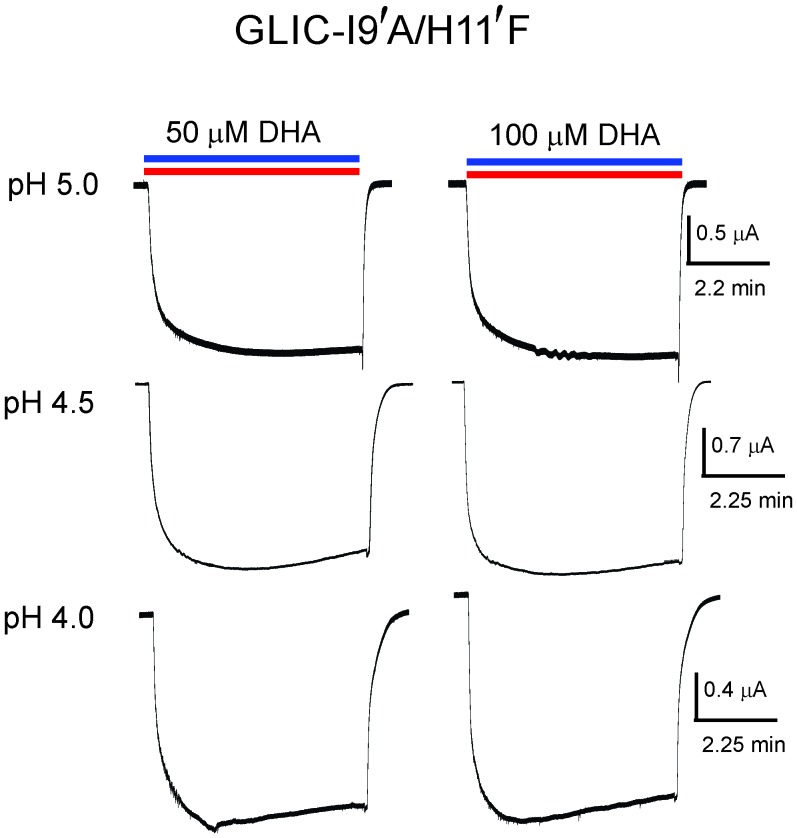
Lack of DHA effect on the non-desensitizing GLIC I9′A/H11′F mutant. Macroscopic currents measured by TEVC for GLIC I9′A/H11′F in response to pH jumps (from 7.4 to the indicated pH value), at −60 mV holding potential, in the presence of either 50 μM DHA or 100 μM DHA. **DOI:** http://dx.doi.org/10.7554/eLife.23886.007

### Crystal structure of GLIC bound to DHA

**Figure 3.**
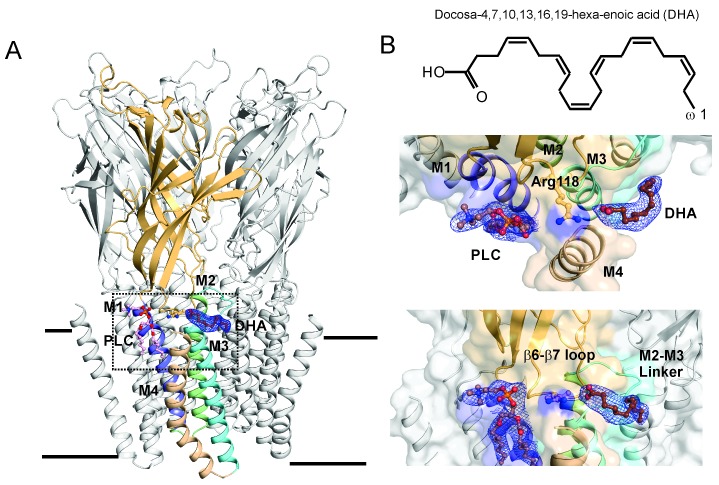
DHA binding site in GLIC. (**A**) A side-view of the GLIC-pH4-DHA structure at pH 4.0 solved to 3.25 Å resolution with a bound DHA molecule shown in stick representation. Only one subunit is colored for clarity (The TM helices are colored as: M1-blue, M2-green, M3-cyan, and M4-wheat). The 2Fo-Fc electron density map for DHA, contoured at 1.0 σ-level, is shown as a blue mesh. The phospholipid molecule (PLC), shown in sticks, was also present in previously reported GLIC structures at acidic pH. (**B**) The chemical structure of the DHA molecule (*top*) and zoomed-in views of the region marked by the inset in panel A (*bottom*). **DOI:** http://dx.doi.org/10.7554/eLife.23886.008

To better understand the mechanism of DHA action and to attempt trapping GLIC in a desensitized conformation, co-crystals of GLIC were grown in the presence of 50 μM of DHA under acidic conditions, similar to those previously reported for GLIC wt (Hilf and Dutzler, 2009). The crystals diffracted up to 3.25 Å and the structure was solved using GLIC-pH4 (PDB ID: 4HFI) as the starting model (Sauguet et al., 2013). Statistics for data collection and refinement are summarized in Table 1. Well-defined electron density was found for DHA in all the subunits in a pocket at the channel periphery; lined by M4, the M2-M3 linker, and the β6-β7 loop (Figure 3A, also see Figure 3—figure supplement 1). The DHA molecule appears bent with a twisted/curled orientation, and makes a salt-bridge interaction with an arginine sidechain (Arg118) in the β6-β7 loop. Besides this interaction, DHA does not appear to engage with the rest of the protein. However, it must be noted that the density for the DHA tail is not resolved beyond C13, and therefore was not built in the model. Considering the high degree of conformational flexibility in the polyunsaturated aliphatic tail of DHA, it is not surprising that this region is less well defined. We therefore cannot exclude additional interactions of the protein with the flexible fatty acid tail. Several residues in the vicinity of the DHA binding site (in the β6−β7 loop, the M2-M3 linker, M1, M3, and M4) show small changes in rotameric orientation (Figure 3—figure supplement 2), although the overall conformation of these regions is similar to the GLIC-pH4 structure. In the GLIC-pH4 structure, the Arg118 side-chain lines a phospholipid-binding pocket within the intra-subunit cavity formed by M1, M3, and M4 (Sauguet et al., 2013). In comparison, the density for the lipid molecule (PLC) in GLIC-pH4-DHA is well-defined in only one subunit and the head-group of this lipid appears to be reoriented in comparison to its position in GLIC-pH4 structure (Figure 3B and Figure 3—figure supplement 2).

**Figure 3—figure supplement 1.**
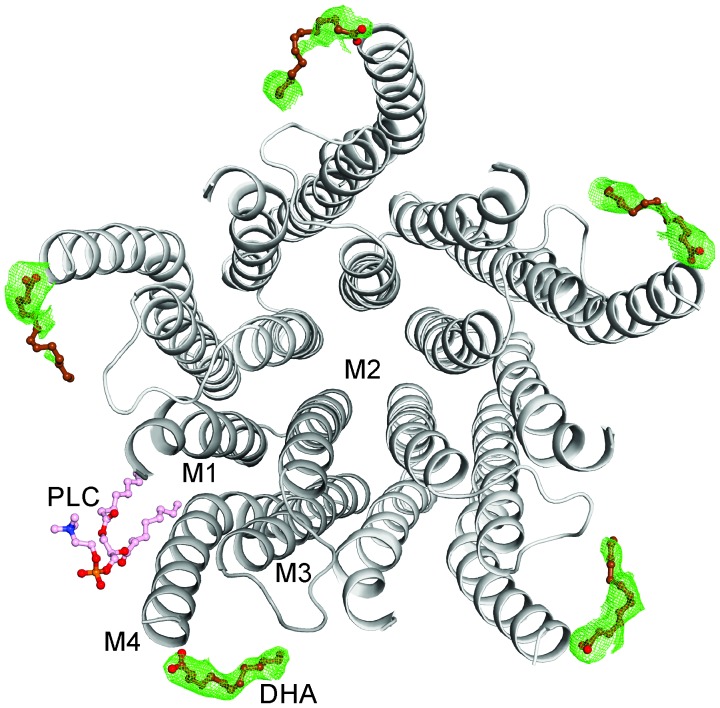
The DHA binding site. The GLIC-pentamer viewed from the extracellular side, overlaid with the F_o_-F_c_ ‘omit’ electron density map generated by excluding DHA molecules from structure factor calculations. The green mesh is contoured at 2.0 σ and DHA molecules are drawn as ball-and-sticks. Prominent electron density is visible for all the five subunits although the continuity was variable among subunits. The lipid molecule (PLC) is also shown in a ball-and-stick presentation. Clear density for the bound PLC was observed at only one subunit. **DOI:** http://dx.doi.org/10.7554/eLife.23886.009

**Figure 3—figure supplement 2.**
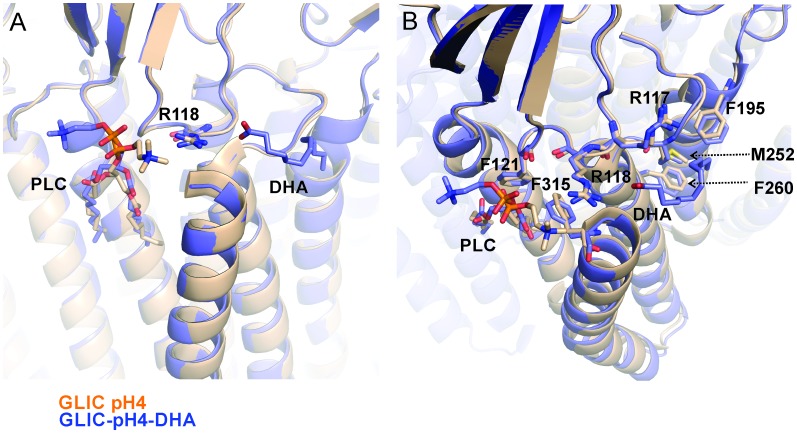
Reorientation of lipid molecule. An alignment of GLIC-pH4 (PDB ID: 4HFI) (Sauguet et al., 2013) and GLIC-pH4-DHA structures shows that the lipid molecule (PLC) bound close to M4 is reoriented. (**A**) A side view of the DHA binding site is shown. (**B**) A top view of the DHA binding pocket. Small changes in side-chain orientation are seen for residues in the β6−β7 loop (Arg117, Arg118, and Phe121), the M2-M3 linker (Met252), M3 (Phe260), M4 (Phe315), and M1 from the adjacent subunit (Phe195). **DOI:** http://dx.doi.org/10.7554/eLife.23886.010

**Figure 3—figure supplement 3.**
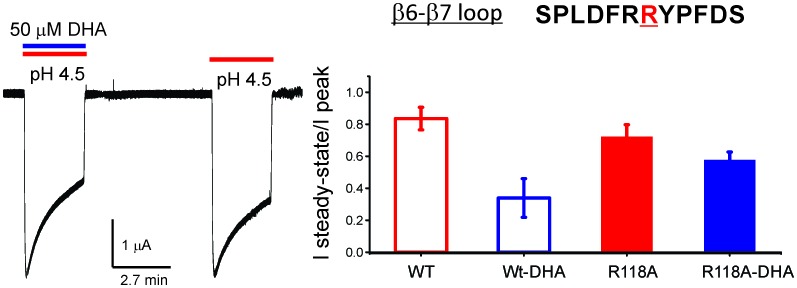
R118A mutation reduces the effect of DHA on desensitization. Sequence for the β6−β7 loop is shown, and the position Arg118 is highlighted in red. Typical GLIC-R118A currents measured by TEVC in response to pH jumps (from 7.4 to 4.5), at −60 mV holding potential, in the presence or absence of 50 μM DHA (*left*). Ratio of the steady-state current (measured at 2.2 min) to the peak amplitude was plotted (n = 12 for GLIC wt and n = 7 for R118A) with s.d shown as error bars (*right*). **DOI:** http://dx.doi.org/10.7554/eLife.23886.011

**Table 1.** Data collection and refinement and statistics of GLIC. **DOI:** http://dx.doi.org/10.7554/eLife.23886.012

Data collection	
Beamline	NE-CAT 24-ID-C/E
Wavelength	0.97870
Space group	C121
Cell dimensions *a, b, c,* (Å); *β* (°)	181.87, 133.32, 159.92; 102.36
No. of observations	198167
No. of unique observations	64340
Resolution range (Å)	61.31–3.25 (3.34–3.25)
CC_1/2_ = 0.3 (Å)*	3.25
Mean *I/σ (I)*	6.7 (1.4)
*R_pim_*^†^	0.057 (0.439)
Completeness (%)	96.7 (98.6)
Multiplicity	2.8 (2.8)
Average Mosaicity	0.49
**Refinement**	
Resolution (Å)	30.0–3.25
*R_work_* (%)	23.15
*R_free_*^‡^ (%)	26.11
**B-factor** (Å^2^)	
Protein	102.55
R.M.S deviations:	
Bond lengths (Å)	0.006
Bond angles (°)	1.45
Molprobity Score	98^th^ percentile
**Ramachandran Analysis^§^**	
Favored	88.15%
Allowed	11.32%
Generously Allowed all Allowed^8^	0.53% (8 residues)

*CC_1/2_ is the Pearson correlation coefficient of two-half data sets (Karplus et al., 2012).

^†^R_pim_ (all I+/I-).

^‡^5.0% of reflections were excluded from refinement for calculation of R_free_.

^§^Calculated using PROCHECK (Laskowski et al., 1993).

To determine if the interaction of DHA with Arg118 is necessary for the observed effect on channel gating, we probed the functional consequence of mutating Arg118 to Ala, and studied the effect of DHA on R118A desensitization. The R118A mutant showed robust pH-induced currents in oocytes, however in comparison to GLIC wt, the current decay was much less affected by 50 μM DHA (Figure 3—figure supplement 3). This finding thereby validates the crystallographically-captured DHA-binding site, and further implicates a novel role for the Arg118 in lipid-channel interactions.

**Figure 4.**
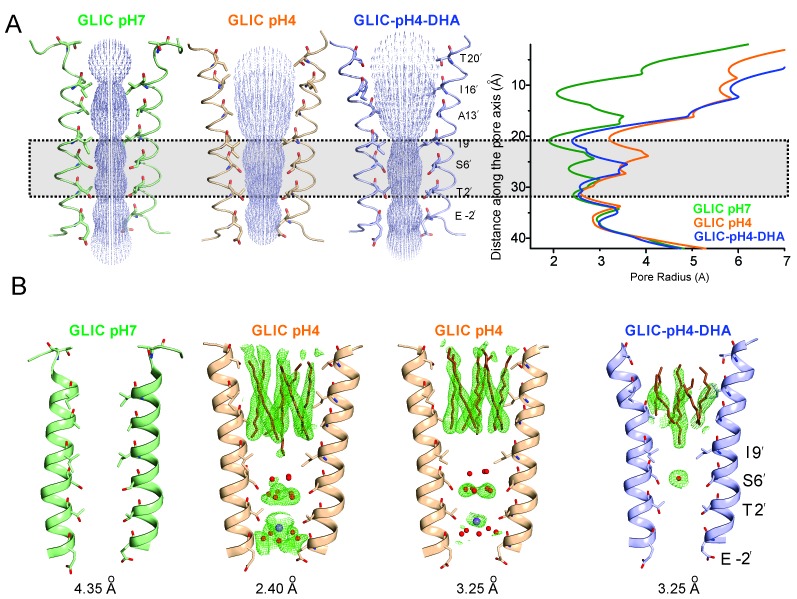
Conformational changes in the GLIC pore. (**A**) The ion-permeation pathway through the channel pore, as determined by the MOLE PyMOL plugin (Petrek et al., 2007). The M2 from two subunits are shown using a ribbon representation, with residues lining the pore represented as sticks (*left*). Pore radius along the channel axis in GLIC structures at pH 7.0 (PDB ID: 4NPQ), pH 4.0 (PDB ID: 4HFI), and at pH 4.0 in the presence of DHA calculated using HOLE software (Petrek et al., 2007; Smart et al., 1996) (*right*). The constricted region from Ile9′ to Thr2′ is highlighted by a grey box. (**B**) F_o_-F_c_ omit electron density of six dodecyl-maltoside molecules (shown in stick representation) and water pentagon (shown as red spheres) at 3.0 σ-level in M2 for different GLIC structures. The PDB ID for the structures are: GLIC-pH7: 4NPQ; GLIC-pH4: 4HFI; GLIC-pH4 (lower resolution): 3UU8. The resolution for each of the structures is indicated below. **DOI:** http://dx.doi.org/10.7554/eLife.23886.013

The most prominent difference noted in the GLIC-pH4-DHA structure in comparison to other GLIC structures (at pH 7.0 and pH 4.0), is at the level of M2 lining the channel pore (Figure 4). The conformation of the GLIC-pH4-DHA pore does not align with either the resting (GLIC-pH7) (Sauguet et al., 2014) or the putative open state (GLIC-pH4) (Sauguet et al., 2013) GLIC structures (Figure 4—figure supplements 1 and 2). In this new M2 conformation, the pore is funnel-shaped with the hydrophobic extracellular-end (Ala13′ to Thr20′) wide-open to a pore radius greater than 5 Å, reminiscent of the GLIC-pH4 structure, while the polar intracellular-half (between Ile9′ and Thr2′) is constricted to 2.5 Å, resembling the GLIC-pH7 closed structure (Figure 4A and Figure 4—figure supplement 4A). The pore radius at the Glu-2’ position was essentially the same as seen in GLIC-pH7 and GLIC-pH4. The F_o_-F_c_ omit map for the M2/M2-M3 linker indicates that the GLIC-pH4-DHA structure is not a mixture of closed and open conformations in the crystal (Sauguet et al., 2014) (Figure 4—figure supplement 3). Further, the observed M2 conformation in GLIC-pH4-DHA is also different from the previously reported locally-closed GLIC structures at pH 4.0 (Prevost et al., 2012) (Figure 4—figure supplement 4B), and therefore represents a unique pore conformation.

**Figure 4—figure supplement 1.**
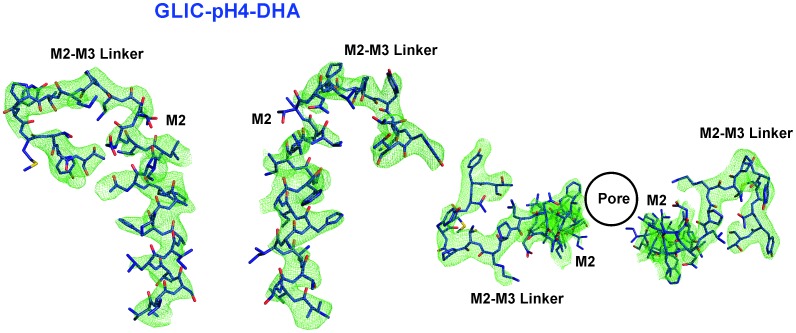
M2 conformation in the GLIC-pH4-DHA structure. Fo-Fc ‘omit’ electron density map (green mesh, contoured at 2.3 σ) for the M2 and M2-M3 linker (228-255) of the GLIC-pH4-DHA structure (shown in stick representation). Two non-adjacent opposed subunits are shown for clarity from the side and top views. **DOI:** http://dx.doi.org/10.7554/eLife.23886.014

**Figure 4—figure supplement 2.**
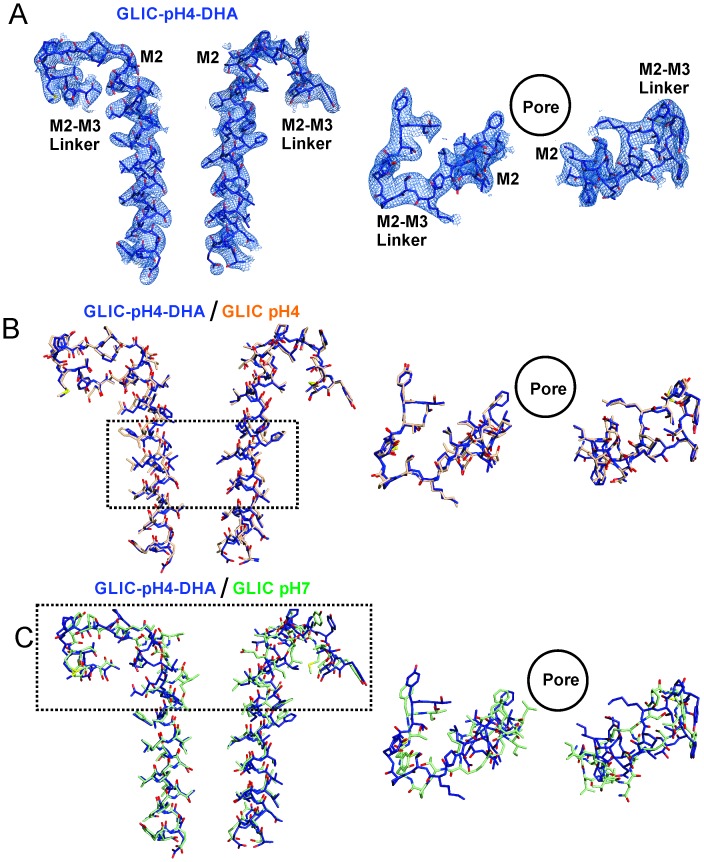
Conformational changes in the GLIC-pH4-DHA structure. The 2F_o_-F_c_ electron density map for the GLIC-pH4-DHA structure contoured at 1.5 σ-level and shown for two non-adjacent subunits. (**A**) Views of M2 and the M2-M3 linker parallel to the membrane (*left*) and from the extracellular end of the membrane (*right*) for the GLIC-pH4-DHA structure. (**B**) The GLIC-pH4-DHA structure aligned with the GLIC-pH4 structure (PDB ID: 4HFI) (Sauguet et al., 2013). (**C**) The GLIC-pH4-DHA structure aligned with the GLIC-pH7 structure (PDB ID: 4NPQ) (Sauguet et al., 2014). **DOI:** http://dx.doi.org/10.7554/eLife.23886.015

**Figure 4—figure supplement 3.**
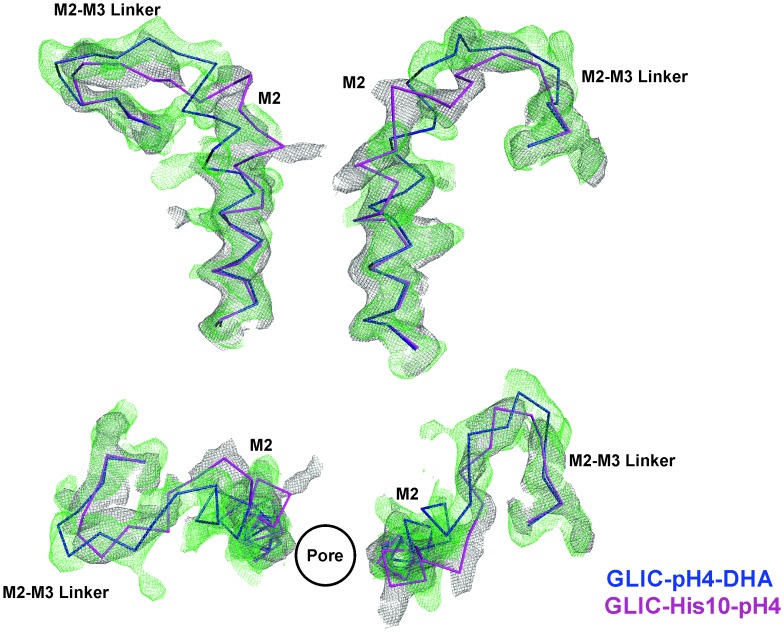
Comparison of Fo-Fc omit maps for the GLIC-pH4-DHA with that of the GLIC-His10-pH4 structure. F_o_-F_c_ ‘omit’ electron density map (green mesh, contoured at 2.4 σ) for the M2 and M2-M3 linker (228-255) of the GLIC-pH4-DHA structure (blue, ribbon representation) overlaid with the F_o_-F_c_ map (grey mesh, contoured at 2.4 σ) for the M2 and M2-M3 linker (228-255) of the GLIC-His10-pH4 structure (PDB ID: 4NPP) (Sauguet et al., 2014) containing a mixture of locally-closed and open conformations. Magenta, ribbon representation shows the locally-closed conformation in 4NPP. Two non-adjacent subunits are shown for clarity from the side and top views. **DOI:** http://dx.doi.org/10.7554/eLife.23886.016

**Figure 4—figure supplement 4.**
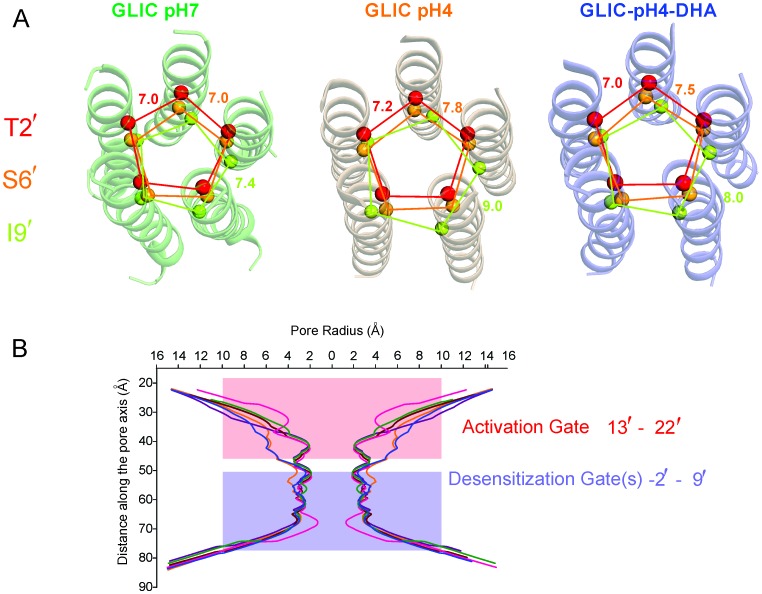
Pore conformations in GLIC structures. (**A**) Cα-Cα distance for the residues lining the narrow constrictions in the GLIC-pH7, GLIC-pH4, and GLIC-pH4-DHA structures (**B**) Pore radius calculated using HOLE (Smart et al., 1996) for GLIC-pH7 (PDB ID: 4NPQ, *green*), GLIC-pH4 (PDB ID: 4HFI, *orange*), GLIC-pH4-DHA (*blue*), and various locally-closed GLIC structures (PDB ID: 3TLS, *purple*; PDB ID: 3TLT, *wine*; PDB ID: 3TLV, *pink*) (Prevost et al., 2012). Residues Leu22′- Ala13′ form narrow constrictions at the extracellular activation gate. The Ile9′ position and the residues below, through the intracellular end, contribute to the desensitization gate(s). **DOI:** http://dx.doi.org/10.7554/eLife.23886.017

The reduction in the GLIC-pH4-DHA pore radius is accompanied by notable changes in the occupancy of detergent molecules, water, and ions within the pore. While the electron density was observed for the bundle of six dodecyl-maltoside molecules in the upper M2, they were more disordered in comparison to the GLIC-pH4 structures (Figure 4B). The reduced detergent occupancy is expected, due to the effect of pore constriction at Ile9′. Additionally, the channel pore in the GLIC-pH4 structure (PDB ID: 4HFI) reveals a cation binding site (at the level of Thr2′ coordinated by water molecules beneath it) and an ordered pentagonal-ring of water molecules that are in-plane with the γ-O atoms of Ser6’ (Sauguet et al., 2013) (Figure 4B). These densities were also noted in lower-resolution structures (comparable in resolution to GLIC-pH4-DHA), although they appeared more diffuse (Hilf and Dutzler, 2009; Bocquet et al., 2009). In contrast, the GLIC-pH4-DHA structure shows a distinctly different hydration profile at the intracellular half of the pore, with loss of densities for water and ions. Interestingly, the orientation of Ser6 sidechains is implicated in directly influencing the organization of the water ring at this position (Sauguet et al., 2013). A small change at this position in GLIC-pH4-DHA brought about by the compression at Ile9’ may thus be responsible for the loss of ions and water.

With the exception of changes in M2, GLIC-pH4-DHA adopts a conformation almost identical to GLIC-pH4 (RMSD for alignment of ECD pentamer (residues 5–191) with 4HFI is 0.34 Å and that for the TMD (residues 192–315) is 0.49 Å). Surprisingly, DHA had minimal effect on the protein conformation in and around M4. Similarly, crystal structures of GLIC at neutral and acidic pH also reveal minimal positional differences in M4 (Figure 5—figure supplement 1). This is somewhat unexpected considering that M4 plays a role in relaying modulatory effects of lipid-protein interactions on to the channel pore, and that M4 perturbations have functional consequences (Bouzat et al., 1998; Lasalde et al., 1996; Lee et al., 1994; Mitra et al., 2004). The lack of a structural change could potentially result from the absence of a membrane environment in crystallographic conditions, with the effect being most pronounced on the lipid-exposed M4 segment. Additionally, crystal packing and lattice forces may overwhelm the energetics of conformational equilibrium, thereby masking the effect induced by ligands and mutations (Gonzalez-Gutierrez et al., 2012). To better understand the role of M4 in regulating lipid-sensitive gating, it is important to first know how M4 moves during channel activation and desensitization.

### Conformational changes in M4 during transition to the ligand-induced desensitized state

To probe the conformational changes in M4 during the transition to the desensitized state, we used site-directed spin labeling (SDSL) and Continuous-Wave (CW) EPR spectroscopic methods in membrane-reconstituted GLIC. Single-cysteine mutations were made along the length of the segment (~31 positions) on a cysteine-less background template. Previous studies have also shown that M4 mutations are well-behaved, with the exception of Pro300, where no pH-activated currents were observed (Carswell et al., 2015; Hénault et al., 2015) Individual cys-mutants were purified, labeled with MTSL, and reconstituted into asolectin membranes for EPR studies. As with any study involving side-chain perturbations, attachment of spin-probes could lead to functional alterations of the channel. We therefore tested the functionality of representative purified cysteine mutants upon spin-labeling and reconstitution by patch-clamp recordings of excised membranes (Figure 5—figure supplement 2). Although the spin-labeled M4 mutants exhibit pH-activated currents, the current traces show qualitative differences in decay profiles. These differences in desensitization kinetics may arise from cys mutagenesis and/or SDSL. Functional perturbation accompanying SDSL remains a caveat of this approach. However, since EPR measurements are made at steady-state conditions, the changes in ‘faster’ components of kinetics may not significantly impact the overall interpretation.

**Figure 5.**
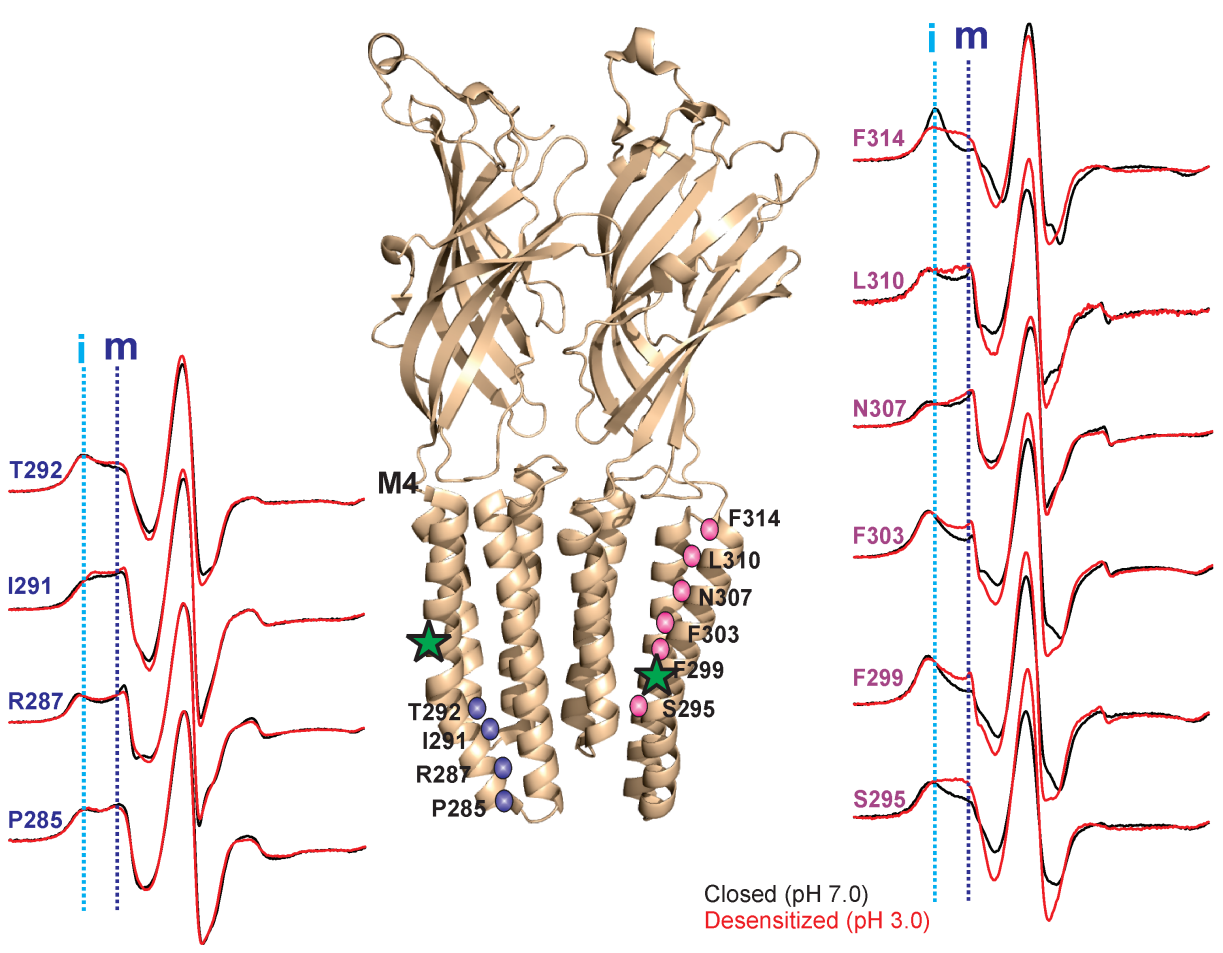
pH-dependent conformational changes in M4 for membrane-reconstituted GLIC. Spin-normalized CW-EPR spectra for representative positions along M4 in the closed (black, pH 7.0) and desensitized (red, pH 3.0) states. Location of spin-labels are shown on the GLIC-pH4 structure (PDB ID: 4HFI). Only two subunits are shown for clarity. The position of Pro300, which introduces a kink in the helix, is marked by a green star. Positions close to and above Pro300 (295-298) show an increase in ΔH_o_^−1^ in the desensitized state (marked by magenta balls), while positions further below are essentially unchanged in the two conformations (marked by blue balls). Dotted lines marked as ‘i’ (cyan) and ‘m’ (dark blue) represent the immobile and mobile components of the spectra, respectively. The two components may arise from two different rotameric orientations of the spin-labels and/or from two conformational states of the protein in equilibrium. **DOI:** http://dx.doi.org/10.7554/eLife.23886.018

The CW spectral analysis included the determination of two parameters: (1) ΔH_0_^−1^, which is measured as the inverse of the central linewidth. This lineshape parameter has been routinely utilized to assess changes in mobility. However, in some cases changes in ΔH_0_^−1^ may arise from alterations in oxygen accessibility that result in line broadening and may not reflect a change in mobility. Nevertheless, this lineshape parameter is useful to reflect changes occurring among the states. (2) Π, accessibility to either membrane or water measured in the presence of lipid-soluble oxygen (O_2_) and water-soluble Ni(II) ethylenediaminediacetic acid (NiEDDA), respectively. Each of these measurements was made for samples at pH 7.0 and at pH 3.0. Since in membranes, exposure to acidic pH activates and subsequently desensitizes GLIC, under steady-state EPR conditions, the channels are expected to be predominantly in their closed conformation at pH 7.0 and in their desensitized conformation at pH 3.0 (Velisetty et al., 2012). An overlay of the EPR line-shapes in the two states for all of the positions studied are shown in Figure 5 and Figure 5—figure supplement 3.

**Figure 5—figure supplement 1.**
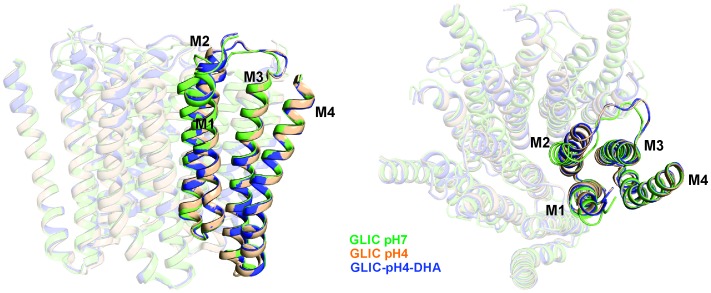
Alignment of GLIC transmembrane domains (TMD). A superposition of GLIC-TMDs (GLIC-pH7, PDB ID: 4NPQ; GLIC-pH4, PDB ID: 4HFI; and GLIC-pH4-DHA) shows minimal conformational differences in the M4 segment. **DOI:** http://dx.doi.org/10.7554/eLife.23886.019

**Figure 5—figure supplement 2.**
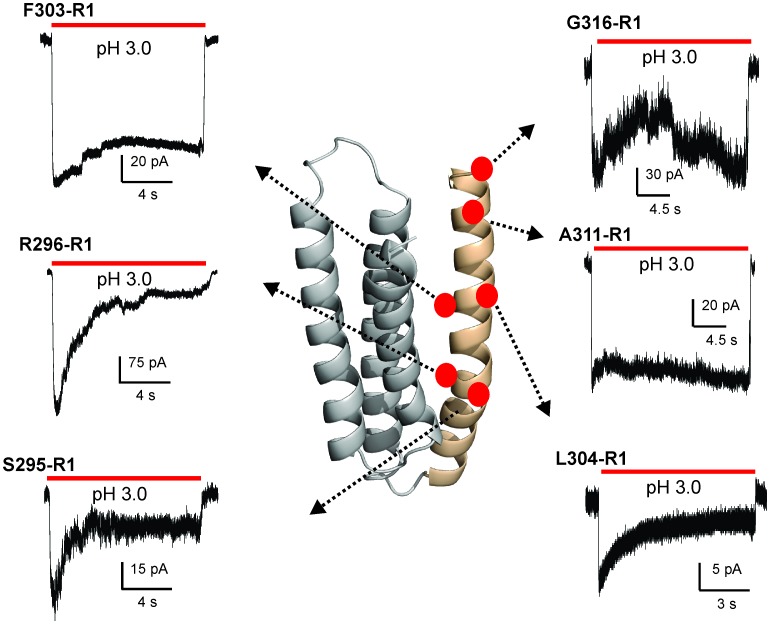
Functional characterization of spin-labeled M4 mutants by patch-clamp recordings in reconstituted proteoliposomes. Macroscopic current traces from ‘inside-out’ patches of representative spin-labeled M4 mutants reconstituted into asolectin membranes. Currents were recorded in response to fast application of pH-jumps from 7.0 to 3.0. Currents were recorded under symmetrical 150 mM Na^+^ at −50 mV holding membrane potential. **DOI:** http://dx.doi.org/10.7554/eLife.23886.020

**Figure 5—figure supplement 3.**
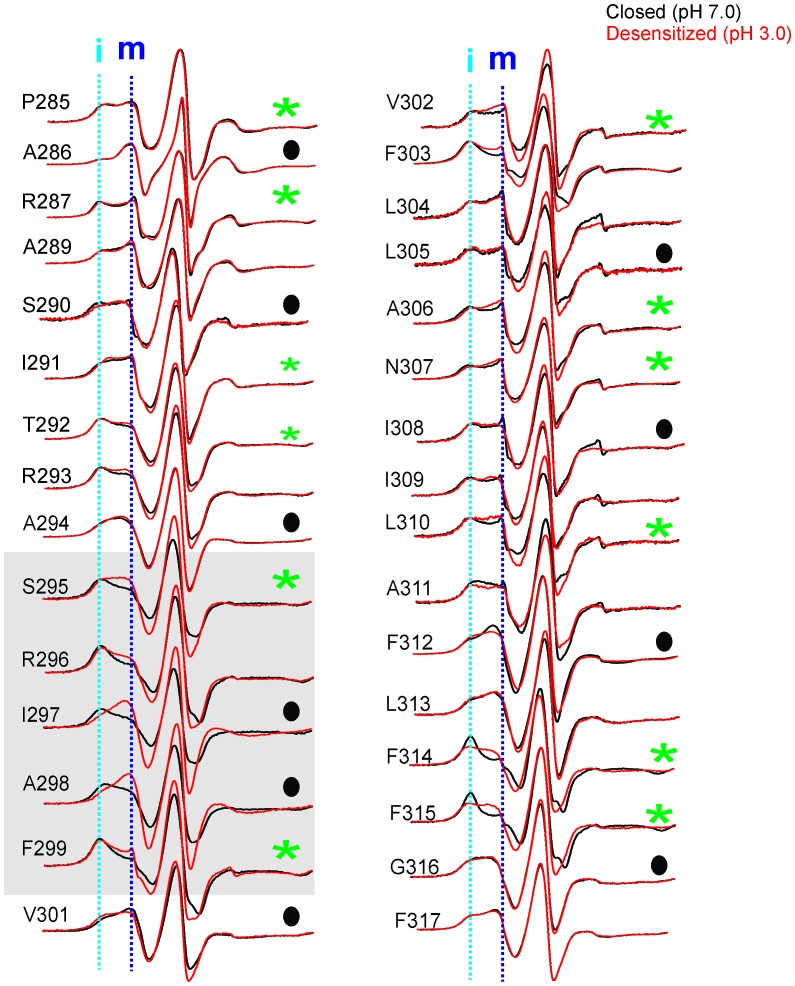
Conformational changes in M4 reported by EPR lineshapes. CW-EPR spectra for the M4 residues in the closed (pH 7.0, black) and desensitized (pH 3.0, red) states. The residues facing the intrasubunit cavity are highlighted by green asterisks and those facing the membrane are indicated by black circles. Dotted lines marked as ‘i’ and ‘m’ represent the immobile and mobile components of the spectra, respectively. Grey box highlights residues at positions (*i-5*) to (*i-1*) with respect to Pro300 (*i = 0*). **DOI:** http://dx.doi.org/10.7554/eLife.23886.021

In the closed state, the spectra at positions lining the TMD-facing interface of M4 (Figure 5, and marked by green asterisks in Figure 5—figure supplement 3) are broad in comparison to the spectra at positions along the membrane-facing M4 interface (marked by black circles in Figure 5—figure supplement 3), suggesting that residues on the TMD-facing interface are packed in a sterically constrained environment. In the desensitized conformation, spectra at the intracellular end (Pro285-Ala294) show minimal changes in lineshape. However, there are dramatic changes in lineshapes for residues above Ala294 that are reflective of an increase in mobility (particularly Ser295, Arg296, Ile297 and Ala298, *grey* box in Figure 5—figure supplement 3; compare the dark blue and cyan dotted lines that indicate the mobile and immobile components of the spectra, respectively). The flexibility in this region is likely due to the hinge/kink introduced by the conserved Pro300 (Figure 5, marked by a green star), arising from steric hindrance of the sidechain and the loss of the backbone hydrogen bonds between Pro300 and Arg296. Proline-induced local distortion of the helix and increases in flexibility are predicted to have global effects on protein conformational changes (Cordes et al., 2002). Consistent with this idea, mutational perturbation of the conserved proline side-chain leads to non-functional channels (Hénault et al., 2015), suggesting that the helix-bending at this position may play a role in channel gating. Above this region (towards the extracellular end), for the TMD-facing side of M4, a large change in lineshape (observed as a decrease in spectral broadening and an increase in amplitude; also see the changes in mobile and immobile spectral components marked by the dotted lines) is noted at several positions (Phe299, Val302, Phe303, Phe314, and Phe315) and modest changes are observed at others (Ala306, Asn307, and Leu310). In contrast, for the membrane-facing side of M4, the spectral changes are small (Figure 5—figure supplement 3, indicated by black circles), indicating that channel gating is accompanied by rearrangement of the packing-interface of the intra-subunit cavity.

**Figure 6.**
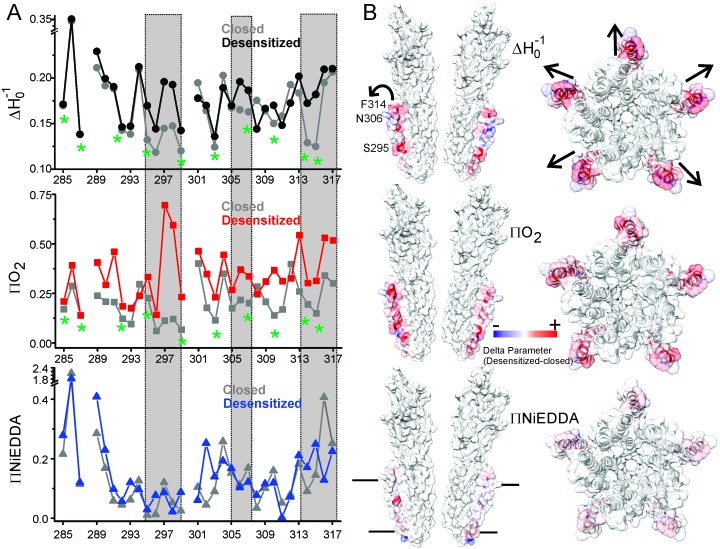
Solvent accessibility changes in M4 during desensitization. (**A**) A plot of residue environmental parameters for the closed (shown in grey) and desensitized (shown in color) states. ΔH_o_^−1^ parameter (*top*); O_2_ accessibility ΠO_2_ (*middle*); water accessibility ΠNiEDDA (*bottom*). Positions along the protein-facing side of M4 are marked by green asterisks. Regions of most prominent change are highlighted within grey boxes. (**B**) Difference in individual parameters between the desensitized and closed states are mapped on the GLIC-pH4 structure (PDB ID: 4HFI) and color-coded with red denoting an increase and blue representing a decrease in the environmental parameter. The direction of putative M4 motion is indicated by the arrows. The putative membrane boundaries as reflected by NiEDDA accessibility are marked by solid black lines in the bottom panel. **DOI:** http://dx.doi.org/10.7554/eLife.23886.022

A complete plot of ΔH_0_^−1^ and accessibility parameters for M4 residues is presented for the closed and desensitized conformations (see Materials and methods for details on the estimation of these parameters), and the difference in values of individual parameters between the two states are mapped on the GLIC-pH4 structure (Figure 6). In the closed conformation, positions with low values of ΔH_0_^−1^ appear to be three to four residues apart and line up on the inward-face of M4 (marked by green asterisk, Figure 6A). Further, high ΠO_2_ values for the membrane-exposed residues and low values for the tertiary contacts show that M4 is closely associated with the rest of the TM helices, such that one face of M4 is protected from lipids in this state. In the desensitized conformation, there is an overall increase in ΠO_2_, which indicates an increase in the membrane exposure of M4. Although, potential differences in membrane permeability of O_2_ in the two pH conditions could affect this measurement. Nevertheless, in comparison to the membrane-facing residues, a larger increase in ΠO_2_ is observed for the TMD-facing residues (Ala289, Ile291, Ser295, Phe299, Val302, Phe303, Ala306, Asn307, Leu310, Phe314, and Phe315) (Figure 6B), suggesting that the intimate association of M4 with M3 and M1 helices is disrupted in the desensitized conformation. Consistent with the observed lineshape changes of residues in the vicinity of the Pro300 kink (295-299), these positions also show high ΠO_2_ values in the desensitized state. Overall, the C-terminal half of M4 shows the most dramatic changes in the CW lineshapes, which are correlated with increases in ΠO_2_. Further, the membrane boundaries of M4 are clearly defined by high ΠNiEDDA values at the extracellular and intracellular end with little water penetration observed in the M4 vicinity. Besides a decrease in water exposure at the C-terminal tip of M4, no major differences are observed in the ΠNiEDDA pattern in the two conformations. A lack of NiEDDA accessibility for the residues facing the intra-subunit cavity is inline with the hydrophobic nature of this region.

**Figure 6—figure supplement 1.**
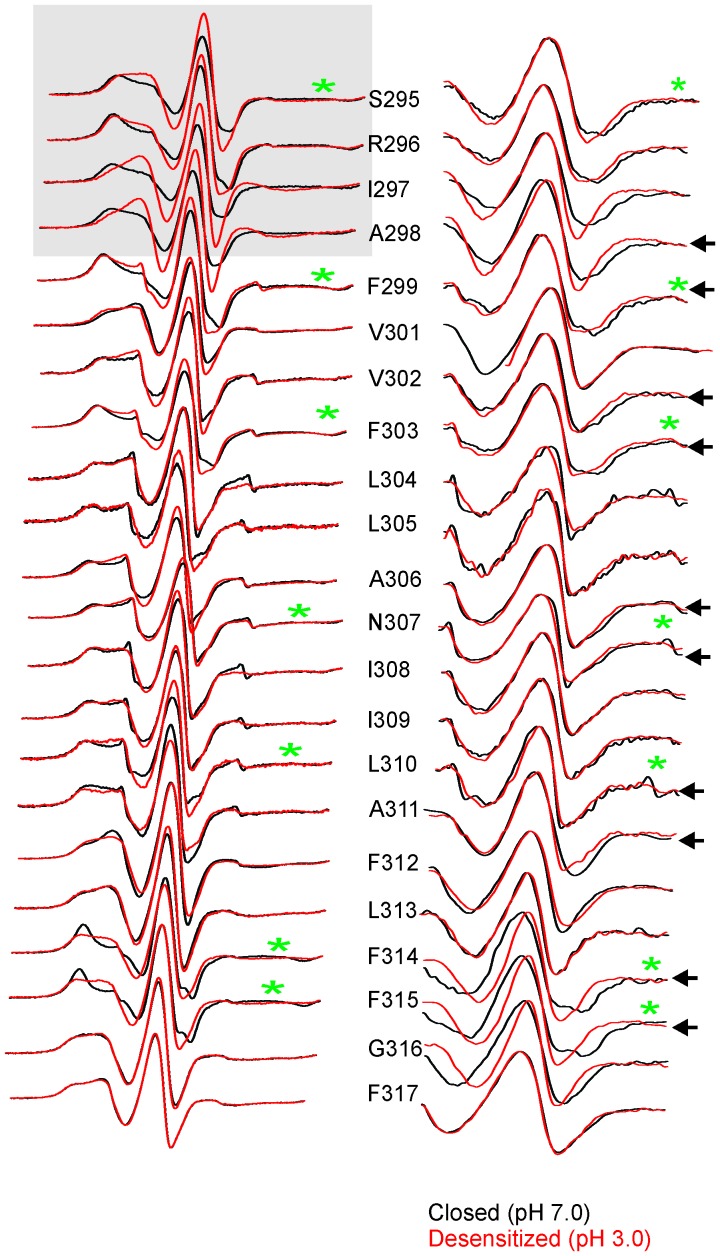
Changes in EPR lineshapes due to relaxation broadening vs mobility changes. CW-EPR spectra for positions along M4 in the closed (pH 7.0) and desensitized (pH 3.0) states before (left) and after purging N_^2^_ (for 15 min) (right). Positions of notable change are marked by black arrows. Positions on the TMD-facing side of M4 are marked green asterisks (these residues are highlighted in the GLIC-pH4 structure as a stick representation). The grey box highlights positions in the vicinity of Pro300 that show large changes in linehapes under the two conditions. **DOI:** http://dx.doi.org/10.7554/eLife.23886.023

Overall, residues facing the intra-subunit cavity show a change in nitroxide lineshapes and increased accessibility to O_2_ reflective of an outward motion of M4 that is accompanied by increased lipid exposure (indicated by the arrows in Figure 6B). Notably, a decrease in water exposure accompanied by an increase in membrane accessibility at the M4 tip is also consistent with an outward M4 motion that would bury the tip further within the membrane.

**Figure 7.**
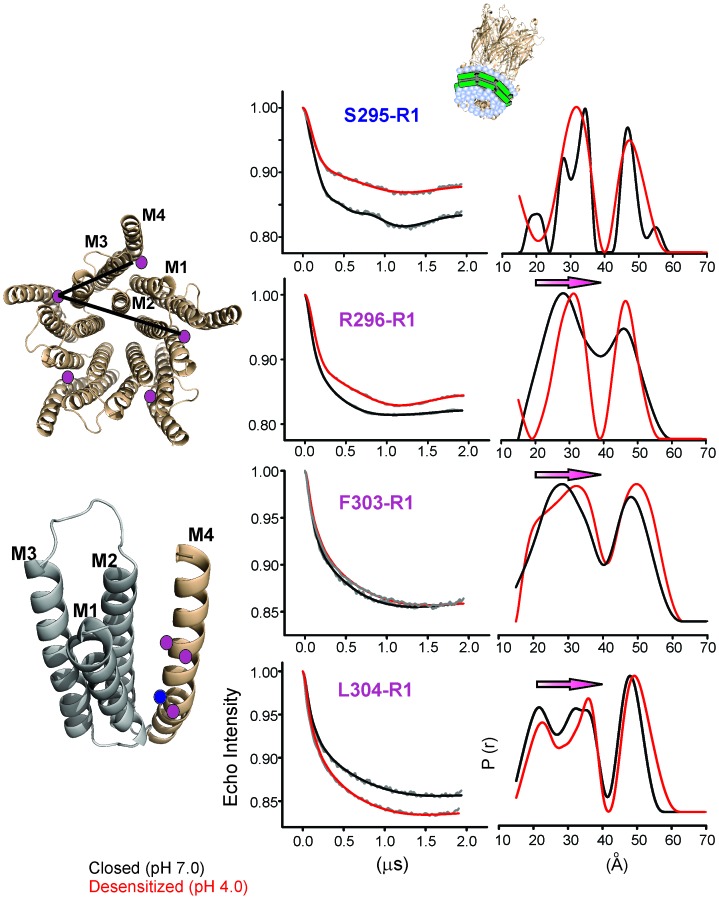
Changes in M4 distance measured by DEER for GLIC in nanodiscs. GLIC structure showing the positions investigated by DEER and the two expected distance distributions (from the adjacent and non-adjacent subunits). Background subtracted DEER- echo intensity is plotted against evolution time and fit using model-free Tikhonov regularization. The corresponding inter-spin distance distribution (right) for the closed (black, pH 7.0) and desensitized (red, pH 4.0) states for different spin-labeled positions. The arrows highlight the direction of change. **DOI:** http://dx.doi.org/10.7554/eLife.23886.024

To quantify the extent of M4 movement, we measured average interspin distances in the closed and desensitized conformations for several positions in M4 (Ser295, Arg296, Phe303, and Leu304) with double electron-electron resonance (DEER) experiments. Although CW spectra suggest a smaller conformational change compared to the C-terminal end, these positions were chosen because they were closer to the distance range that can be reliably measured by DEER (20–60 Å). Since DEER measurements of longer distances are particularly challenging to determine in liposomes, we reconstituted labeled GLIC in nanodiscs using the membrane scaffolding protein, MSP1E3D1 (Ritchie et al., 2009). Previous work has shown that the use of nanodisc technology in combination with Q-band for data collection improved DEER sensitivity by lowering background contributions (Zou and McHaourab, 2010). As expected for a pentameric system with five labels, the distance distribution for all the positions showed at least two main components, one corresponding to the short adjacent distance and the other to the long non-adjacent distance (Figure 7, Figure 7—figure supplement 1). The DEER distances in the closed state were broadly consistent with the GLIC-pH7 structure (PDB ID: 4NPQ) (Table 2). Positions S295R1 and L304R1 also reveal multiple components for the adjacent distances indicative of conformational heterogeneity of the M4 helix. However, the DEER distances in the desensitized state were consistently longer (for three of four positions) than those predicted from the GLIC-pH4 structure (PDB ID: 4HFI) and the GLIC-pH4-DHA structure. For positions Arg296, Phe303, and Leu304, both distributions move toward longer distances in the desensitized state compared to the closed state. Increases in DEER distances are a consequence of M4 moving away from the five-fold axis, up to 4 Å at the mid-M4 region and likely to be greater at the tip of M4. Based on CW and DEER measurements, we conclude that M4 undergoes major conformational changes during its transition to the ligand-activated desensitized state, with an increase in lipid exposure of this segment, particularly at positions along the protein-facing side of M4.

**Figure 7—figure supplement 1.**
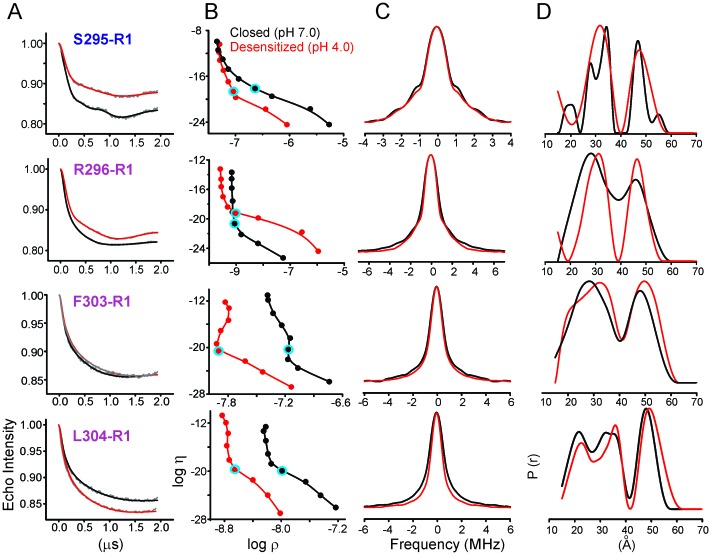
Analysis of DEER data. (**A**) Background corrected Q-band dipolar evolution data for spin-labeled GLIC mutants reconstituted in nanodisc. (**B**) Corresponding Tikhonov L-curve. The highlighted blue circle in the L-curve represents the regularization parameter (α = 100) corresponding to the distance distribution in panel D. (**C**) The FT spectra of the traces shown in panel A. The black and red traces are fits based on the distance distribution shown in panel D for samples in the closed (pH 7.0) and desensitized (pH 4.0) states, respectively. (**D**) Distance distribution obtained by Tikhonov regularization. **DOI:** http://dx.doi.org/10.7554/eLife.23886.025

**Table 2.** DEER distances measured in nanodiscs compared with (c_β_-c_β_) distances from crystal structures. **DOI:** http://dx.doi.org/10.7554/eLife.23886.026

		**pH 7.0**		**pH 4.0**	
**Residue**		**Short (Å)**	**Long (Å)**	**Short (Å)**	**Long (Å)**
**S295R1**	GLIC Crystal Structure	26.3	42.6	26.5	42.9
	**GLIC Nanodisc DEER**	27.8/34.3	46.6	31.6	47.2
**R296R1**	GLIC Crystal Structure	28.0	45.3	28.2	45.7
	**GLIC Nanodisc DEER**	27.8	46.1	31.1	46.7
**F303R1**	GLIC Crystal Structure	29.2	47.3	29.5	47.8
	**GLIC Nanodisc DEER**	28.1	48.3	32.2	49.4
**L304R1**	GLIC Crystal Structure	34.5	55.9	35.3	57
	**GLIC Nanodisc DEER**	21.4/33.8	47.8	22.6/35.6	49.4

GLIC crystal structure at pH 7.0 (PDB ID: 4NPQ) (Sauguet et al., 2014).

GLIC crystal structure at pH 4.0 (PDB ID: 4HFI) (Sauguet et al., 2013).

**Figure 8.**
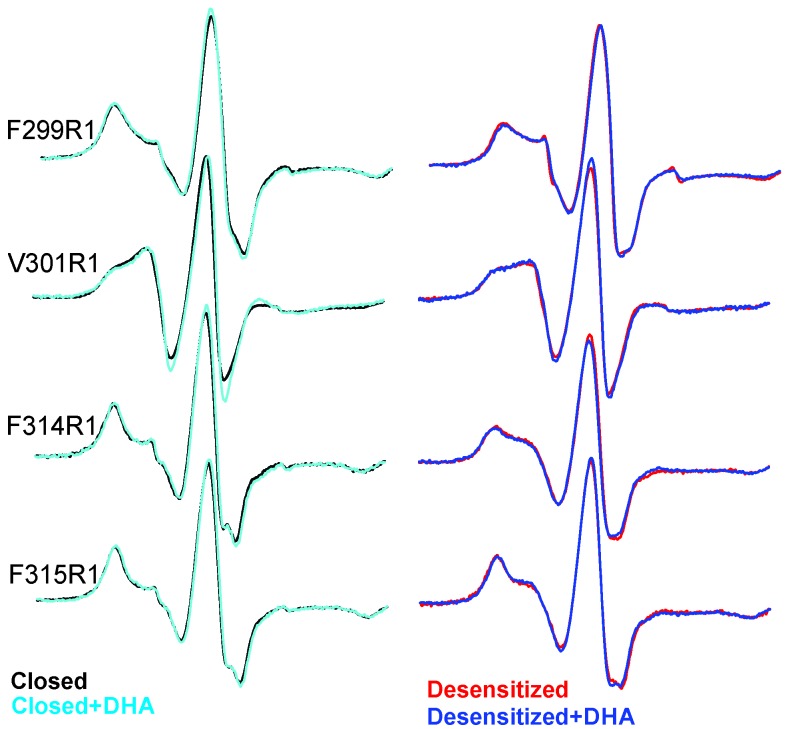
EPR spectral analysis of M4 positions in the absence or presence of DHA. Spin-normalized CW-spectra for representative positions along M4 in the closed (pH 7.0) and desensitized (pH 3.0) states and measured in the presence or absence of 50 μM DHA. **DOI:** http://dx.doi.org/10.7554/eLife.23886.027

Outside of the canonical gating scheme as described in Figure 1A, pLGICs are shown to also exist in a lipid-induced, non-activatable conformation, referred to as the ‘uncoupled’ state (daCosta and Baenziger, 2009; daCosta et al., 2013). This conformational state, which is distinct from the desensitized state, is refractory to agonist-induced transitions and is characterized by lower agonist-affinity (similar to the closed state). To determine if the DHA-stabilized state is similar to the ‘uncoupled’ state, we measured CW-spectra at representative positions on M4 under steady-state conditions of neutral and acidic pH (that favor the closed and desensitized conformations, respectively), each in the presence and absence of DHA (Figure 8). If the observed effect of DHA was through an increase in the rate of agonist-induced desensitization, under conditions of prolonged agonist exposure, the equilibrium population, both in the presence and absence of DHA, is likely to be shifted towards the desensitized state. However, if DHA were to stabilize the uncoupled conformation, then a mixture of closed and uncoupled states, with very little population of the desensitized states, is expected in the acidic pH condition. A comparison of the spectra (Figure 8) shows that there is indeed no effect of DHA on the lineshapes of the two end-states (closed and desensitized). These results further confirm that DHA stabilizes an agonist-induced desensitized state.

## Discussion

The GLIC-pH4-DHA structure presented here reveals a novel lipid-induced conformation in the crystal that is physically distinct from previously observed pLGIC conformations (Du et al., 2015; Sauguet et al., 2014; Hilf and Dutzler, 2009, 2008; Sauguet et al., 2013; Miller and Aricescu, 2014; Hassaine et al., 2014; Hibbs and Gouaux, 2011; Morales-Perez et al., 2016). A comparison of GLIC-pH7 and GLIC-pH4 structures suggest that activation involves an outward tilting of M2 from the pore-axis leading to channel opening with a radius >5 Å at the extracellular end (Hilf and Dutzler, 2009; Sauguet et al., 2013). In the GLIC-pH7 structure, the M2 helices come together to form a tightly packed bundle with the pore constricted to less than ~2.5 Å at Ile16′, Ile9′, Ser6′, and Thr2, effectively occluding ion permeation (the metal-oxygen distance of hydrated monovalent cations is ~2.1–3.1 Å (Hille, 2001; Marcus, 1988). In the GLIC-pH4 structure, the narrowest region of the pore is formed by the selectivity filter region (between Thr2′ to Glu-2′) with a radius ~2.5 Å at Thr2′. Although the pore in this region is too narrow to allow fully-hydrated cations to pass, polar side-chains at Ser6′, Thr2′ and Glu-2′ could potentially coordinate partially hydrated ions. Since it is not known whether GLIC can permeate partially hydrated ions, the conformational state of GLIC-pH4 (open, pre-open, or desensitized) is still debatable. In contrast, in the GLIC-pH4-DHA structure, while the extracellular end remains open, the intracellular-half of M2 (lined by Ile9′, Ser 6′, and Thr2′) undergoes constriction, with the largest effect seen at Ile9′ (pore radii ~2.5 Å). In this conformation, occupancy of the ions and water molecules in the selectivity filter area is greatly reduced, which could be induced by the closure at Ile9′. The pore radii at Glu-2′ is unchanged. However, lower resolution of our structure limits us from making significant interpretations about the side-chain conformation at the −2′ position. We therefore suggest that the GLIC-pH4-DHA structure represents a desensitized conformation (perhaps a lipid-induced, deeper state), where ion permeation is occluded in the intracellular-end of M2 below the 9′ position. Consistent with this idea, reducing the side-chain volume or hydrophobicity at the conserved 9′ position increases the open state stability and decreases the apparent desensitization rate in many members of the pLGIC family (Bocquet et al., 2007; Labarca et al., 1995; Filatov and White, 1995; Akabas et al., 1992; Yakel et al., 1993; Chang et al., 1996; Revah et al., 1991). Further, constriction of the inner half of M2 in the desensitized state was previously predicted based on our EPR spectral broadening information (Velisetty et al., 2012). This mechanism is also consistent with lidocaine slowing desensitization by a ‘foot-in-the-door mechanism’ (Velisetty and Chakrapani, 2012). Other studies have suggested that lidocaine accelerates GLIC desensitization (although these were measured at lower lidocaine concentrations) (Gonzalez-Gutierrez and Grosman, 2015). Pore dehydration and loss of ion occupancy at the selectivity filter region have been implicated in C-type inactivation in voltage-gated channels (Cuello et al., 2010a). Our structure lends strong support to the original ‘two-gate’ hypothesis that suggests the presence of two structurally distinct activation and desensitization gates (Auerbach and Akk, 1998). This model is also consistent with accessibility measurements of the pore lining residues in the three conformations (Wilson and Karlin, 2001). At the functional level, pLGICs are known to display multiple desensitized states with dwell times ranging from milliseconds to minutes (Elenes and Auerbach, 2002). Although, an unequivocal assignment to one of these states is not discernable, prolonged agonist exposure favors a long-lived desensitized state, and we therefore hypothesize that this state may be favored in the crystal form.

Recently, structures of GABA_A_R-β3, GlyR, and nAChR-α4β2 were solved in the presence of agonists and are likely to represent desensitized conformations (Du et al., 2015; Miller and Aricescu, 2014; Morales-Perez et al., 2016). In these structures, a local constriction was noted in the M2 intracellular end, closer to the −2′ position, which is consistent with findings that picrotoxin binding at −2′ slows desensitization (Gielen et al., 2015). Interestingly, the extent of pore closure at −2′ appears to bear some correlation with the desensitization properties of the these channels: a stronger desensitization was observed for GABA_A_R-β3 and nAChR-α4β2 currents (Miller and Aricescu, 2014; Morales-Perez et al., 2016) in comparison to ivermectin/glycine evoked currents for GlyR (Du et al., 2015). In terms of the structures, the pore is narrower in GABA_A_R-β3 and nAChR-α4β2 (~1.5–2 Å radius) compared to GlyR (~2.5 Å radius). Furthermore, in ELIC (a GABA-gated cation channel), the intracellular compression is suggested to also involve residues further up (6′ to −2′ positions) (Kinde et al., 2015). Overall, these mechanistic differences may underlie the broad range of desensitization kinetics observed within the family, encompassing time constants that span several orders of magnitude (<1 ms for α7-nAChR, tens of seconds for GLIC and ELIC, and very little current decay observed for ρ1 GABA_A_R and α GlyR, 5-HT_3A_R) (Keramidas and Lynch, 2013).

A notable aspect of the new conformation reported here is that it is stabilized by a physiologically relevant lipid molecule. The question still remains as to how DHA induces the observed conformational state. The DHA binding-site in the M4 vicinity was not surprising, considering the extensive functional and biophysical studies that have implicated M4 as the ‘lipid-sensor’ in pLGIC. Further, earlier studies predicted that the effect of free PUFA on nAChR inhibition occurs through allosteric mechanisms that alter lipid-protein interactions (Villar et al., 1988; Andreasen and McNamee, 1980; Fernández Nievas et al., 2008) rather than through changes in bulk membrane fluidity. Although the M4 segment is least conserved in sequence, mutations (including those at the lipid-facing side of the helix) have been shown to alter gating, presumably by perturbing the protein structure or the lipid-protein interactions (Bouzat et al., 1998; Lasalde et al., 1996; Li et al., 1992). Similarly, DHA appears to alter the interaction of the channel with an annular lipid molecule (PLC) in the M4 groove and, perhaps as a consequence, exerts a long-range allosteric effect on the pore conformation. Consistent with this idea, the occupancy of PLC in this site appears to be influenced by the conformational state of the channel. Particularly, the GLIC-pH7 and locally-closed GLIC structures show no electron density at this PLC site, suggesting that the open conformation may stabilize the binding of this lipid molecule (Sauguet et al., 2014; Prevost et al., 2012). Further, the most notable consequence of propofol binding in the GLIC structure was the change in the orientation of PLC, suggesting that alterations in channel properties may arise from changes in lipid-protein interactions (Nury et al., 2011). In remarkable agreement, our EPR data show that residues lining the PLC pocket in the crystal structure (302–316 in M4 and the 118–121 in the β6-β7 loop [Velisetty et al., 2014]) show an increase in O_2_ accessibility in the desensitized state, indicating that the lipid-accessibility of this pocket is increased upon activation. However, the pathway for allosteric coupling between the DHA binding site and the pore is not clear, since the conformations of M4, the M2-M3 linker, and the β6−β7 loop are nearly identical to those observed in the GLIC-pH4 structure. One possibility is that this region undergoes minimal structural change between the open and desensitized states. Further studies to stabilize the open GLIC conformation in membranes are needed to fully address this question.

Based on our EPR data, which show that the lipidic environment of M4 changes quite dramatically between the closed and desensitized states accompanied by an increase in intra-subunit distances, we propose that the M4 segment undergoes an outward tilt upon activation, mediated by the hinge/bend at the conserved proline residue in the middle of M4. The structural changes observed in EPR are consistent with the study in GLIC M4 that shows that mutational perturbations in the extracellular half of M4 have a greater effect on pH_50_ in comparison to the intracellular end (Hénault et al., 2015). The proposed M4 conformational change is also consistent with the lipid-mediated M4 tilt observed in isolated peptides (Antollini et al., 2005). Since this proline residue is also present in other members of the family including GABA_A_Rs and GlyRs, the proposed M4 movement could be a conserved mechanism. The outward M4 motion is associated with changes in both the polarity and the volume of the intra-subunit cavity and such a conformational change may underlie state-dependent accessibility of several lipophilic modulators (such as alcohols and anesthetics) of the pLGIC which are shown to bind in these cavities (Mihic et al., 1997; Nury et al., 2011; Howard et al., 2011). Importantly, several residues on the TMD-facing side of M4lining this hydrophobic cavity, are proposed to bind allosteric modulators such as PNU-120596 (Young et al., 2008) and endogenous steroid (THDOC) (Hosie et al., 2006), that modulate desensitization in nAChR and GABA_A_R, respectively. From a structural point of view, it is intriguing that the M4 position appears to be fixed with respect to the rest of the TM helices in the pLGIC structures, even though these structures may represent different conformational states. In contrast, functional analysis of M4 mutations and our EPR data seem to suggest that M4 is dynamic and experiences extensive change in environment during gating. Although we cannot explain this disparity with certainty, it is conceivable that M4 movements could be masked in a detergent environment. It is also possible that the lipidic environment around M4 changes without a significant change in the M4 backbone. We believe that high-resolution structural studies of pLGIC in a membrane environment, such as nanodiscs, are needed to address these differences.

In conclusion, we show that distinct regions of the pore control activation and desensitization in pLGICs, in contrast to the mechanisms proposed in tetrameric ligand-gated channels (Sobolevsky, 2015). Pore dehydration and collapse of the selectivity region during desensitization/inactivation appears to be a conserved mechanism across many channel types (Cuello et al., 2010b). The present work provides a structural view of the long-studied allosteric modulation of lipids on pLGIC desensitization and opens up new avenues for the investigation of more complex regulatory mechanisms.

## Materials and methods

### Cloning and functional measurements in oocytes: 

The gene encoding GLIC was inserted into the pTLN vector for oocyte expression and confirmed by DNA sequencing. The DNA was then linearized with the *Mlu1* restriction enzyme overnight at 37^°^C. The mRNA was synthesized using the mMessage mMachine kit (Ambion, Life Technologies, Carlsbad, CA), purified with RNAeasy (Qiagen, Germantown, MD), and injected (5–15 ng) into *Xenopus laevis* oocytes (stages V-VI). Control ooctyes were injected with the same volume of water to verify endogenous currents were not present. Oocytes were maintained at 18^°^C in OR3 media (Leibovitz media, GIBCO BRL: Life Technologies, Carlsbad, CA) containing glutamate, 500 units each of penicillin and streptomycin, pH adjusted to 7.5, osmolarity adjusted to 197 mOsm). Two electrode voltage-clamp experiments were then performed at room temperature 2–5 days after injection. A Warner Instruments (Hamden, CT) Oocyte clamp OC-725 was used for the measurements, and the current was sampled and digitized at 500 Hz with a Digidata 1440A (Molecular Devices, Sunnyvale, CA). Oocytes were clamped at a holding potential of −60 mV, and current traces were recorded in response to ligand application. Solutions were changed using a syringe pump perfusion system flowing at a rate of 2 ml/min. The electrophysiological solutions contain 96 mM NaCl, 2 mM KCl, 1.8 mM CaCl_2_, 1 mM MgCl_2_, and 5 mM HEPES (pH 7.4, osmolarity adjusted to 195 mOsm) or 5 mM Sodium Citrate (Hilf and Dutzler, 2009; Parikh et al., 2011; Hilf et al., 2010; Goyal et al., 2011) (at acidic pH buffer, pH adjusted to indicated value (4.0–6); osmolarity adjusted to 195 mOsm). All chemical reagents were purchased from Sigma-Aldrich. DHA stock solutions were freshly prepared in DMSO prior to each experiment and diluted to the final concentration in the electrophysiology solutions (*mentioned above*). The highest DMSO concentration used was ~0.016% and comparison with control experiments were made using the highest concentration of DMSO in the test conditions. The traces were analyzed by Clampfit 10.2 (Molecular Devices, Sunnyvale, CA). Dose response curves were fit in Origin (OriginLab, Northampton, MA) to determine the pH_50_ and Hill coefficient (n_H_). The values for ‘n’ in the figure legends referto the number of oocytes.

### Cloning and protein expression

The GLIC gene cloned into a modified pET26b vector was expressed as a fusion construct with N-terminal maltose binding protein (MBP) as previously described (Hilf and Dutzler, 2009; Bocquet et al., 2009). The protein was expressed and purified as previously described (Hilf and Dutzler, 2008; Bocquet et al., 2009; Velisetty and Chakrapani, 2012). Briefly, C43 *E.coli* cells (Lucigen Corporation, Middleton, WI) transformed with the construct were grown in terrific broth media containing 50 μg/ml kanamycin at 37°C to O.D_600_ of 1.0. Cells were induced with 0.2 mM isopropyl 1-thio-β-d-galactopyranoside (Gold Biotechnology, Olivette, MO) overnight at 18°C. Membranes were prepared by homogenizing the cells in Buffer A (100 mM NaCl, 20 mM Tris-HCl (pH 7.4)) with protease inhibitors and centrifuged at 100,000 x g for 45 min. Membranes were solubilized in Buffer A using 40 mM DDM (*n*-dodecyl-β-d-maltopyranoside, Anatrace Inc, Maumee, OH) at 4°C. The protein was purified by binding to amylose resin and eluting with 20 mM maltose. The maltose binding protein tag was cleaved with human rhinovirus 3C protease (GE Healthcare, Wauwatosa, WI), and the pentameric protein was separated from MBP using size exclusion chromatography on a Superdex 20/200 column (GE Healthcare, Wauwatosa, WI) with Buffer A and 0.5 mM DDM.

### Site-directed spin labeling

The native Cys (C27) was mutated to Ser and single Cys mutants in M4 were generated using the Cys-free construct (C27S) as the template. Mutant proteins were expressed and purified similar to the *wild type* channels. Purified mutants were labeled with a methanethiosulfonate spin probe MTSL (1-Oxyl-2,2,5,5-tetramethylpyrrolidin-3-yl) methyl methanethiosulfonate) (Toronto Research Chemicals Inc, North York, ON, Canada) at a 10:1 label:protein molar ratio and incubated on ice for 30 min, after which a 5-fold molar excess of the MTSL was added and further incubated for 2 hr for better labeling efficiency (Velisetty et al., 2012). The labeled protein was then purified by size exclusion chromatography on a Superdex 20/200 column (GE healthcare, Wauwatosa, WI) in Buffer A supplemented with 0.5 mM DDM. Spin-labeled samples were reconstituted at a 1:3000 protein:lipid (molar ratio) in asolectin, incubated with biobeads to remove solubilizing detergent, and centrifuged to obtain a pellet of the proteoliposomes.

### EPR spectroscopy and analysis

Continuous Wave-Electron Paramagnetic Resonance (CW-EPR) spectroscopy measurements were performed at room temperature on an EMX X-band spectrometer (Bruker, Billerica, MA) equipped with a dielectric resonator and a gas permeable TPX plastic capillary. First derivative absorption spectra were recorded at an incident microwave power of 2.0 mW, modulation frequency of 100 kHz, and modulation amplitude of 1.0 G. The EPR signal is normalized to the total number of spins in the sample by dividing the spectra by the peak-to-peak value of the double integral (which is proportional to the total number of spins). Our analyses were centered on two types of dynamic EPR structural information (Farahbakhsh et al., 1992; Altenbach et al., 2005): the first is lineshape parameter ΔH_o_^−1^, calculated as the inverse of the central line width of the first derivative absorption spectra, which often times correlates with the mobility. ΔH_o_^−1^ is governed both by the local steric contacts in the immediate vicinity of the probe and by the flexibility of the backbone to which it is attached (Mchaourab et al., 1996). Lineshape changes at times may not arise from changes in mobility but occur because of relaxation broadening due to accessibility to paramagnetic O_2_. This scenario can be distinguished by measuring lineshapes upon purging N_2_ (See Figure 6—figure supplement 1). For positions where relaxation is observed, we will refer to variations in ΔH_o_^−1^ as lineshape changes rather than differences in mobility. The second is spin-probe solvent accessibility evaluated by collisional relaxation methods. Here, polar Ni(II) ethylenediaminediacetic acid (ΠNiEDDA) and nonpolar molecular O_2_ serve to evaluate the extent of water and membrane exposure, respectively (Farahbakhsh et al., 1992; Gross and Hubbell, 2002). The accessibility parameter (П) is estimated from power saturation experiments in which the vertical peak-to-peak amplitude of the central line of the first derivative EPR spectra is measured as a function of increasing incident microwave power (Farahbakhsh et al., 1992). Conformational changes were measured by equilibrating the sample with appropriate buffers (pH 7.0 and 3.0) in a 42°C water-bath. The samples were centrifuged and the process was repeated three times to ensure complete buffer exchange. These conditions ensured saturation of pH-induced changes in EPR line-shape (Velisetty et al., 2012). Reversibility of structural changes was ensured by switching back to pH 7.0.

### Expression of membrane scaffolding protein and GLIC reconstitution in nanodisc

Membrane scaffold protein (MSP1E3D1) was expressed and purified as previously described (Boldog et al., 2007; Mishra et al., 2014) with some modifications. The MSP1E3D1 gene in pET-28a (a gift from Stephen Sligar: Addgene plasmid # 20066) (Denisov et al., 2007) was transformed in *E. coli* BL21(DE3) cells (Agilent Technologies, Santa Clara, CA) and plated on LB-agar plates supplemented with kanamycin (25 μg mL^−1^). An overnight culture from a single colony was set up with LB supplemented with kanamycin (25 μg mL^−1^) and 1% glucose. The overnight culture was used to inoculate a 1L culture of Terrific broth supplemented with kanamycin (25 μg mL^−1^) and 0.2% glucose. The culture was grown at 37°C with shaking to an OD_600_ of ~1.0, and induced by with 1 mM IPTG for 4 hr at 37°C. Cells were harvested by centrifugation and the cell pellet resuspended in Buffer A containing 1 mM PMSF and Complete EDTA-free protease inhibitor cocktail tablet (Roche) and lysed by homogenization. The lysate was centrifuged at 30,000 x g for for 30 min and the supernatant was bound to Ni-NTA equilibrated with Buffer A. The resin was washed with four bed volumes of Buffer B (300 mM NaCl, 40 mM Tris-HCl, and pH 8.0) containing 1% Triton X-100, four bed volumes of Buffer B containing 50 mM sodium cholate, four bed volumes of Buffer B, four bed volumes of Buffer B containing 20 mM imidazole, and eluted with Buffer B containing 300 mM imidazole. The eluted MSP1E3D1 was passed through a desalting column equilibrated with Buffer C (100 mM NaCl, 50 mM Tris-HCl, 0.5 mM EDTA, and pH 7.5), and the concentration was determined by absorbance at 280 nm (extinction coefficient = 29,910 M^−1^ cm^−1^). The purity was assessed by SDS–PAGE and size-exclusion chromatography.

Detergent-solubilized spin-labeled GLIC mutants passed through gel-filtration columns were incorporated into lipid nanodiscs as previously described (Mishra et al., 2014) with some modifications. Briefly, asolectin dissolved in chloroform was dried using nitrogen stream and rehydrated in Buffer A supplemented with 2 mM DDM. Each spin labeled mutant was mixed with rehydrated lipids and MSP1E3D1 in the GLIC:MSP:lipid = 1:3:360 molar ratio. The mixture was incubated at 4°C for 30 min with gentle rotation. Bio-beads SM-2 (Bio-Rad Laboratories, Hercules, CA) were added to initiate reconstitution overnight at 4°C with gentle rotation. Bio-beads were then removed and the reconstitution mixture assessed by size-exclusion chromatography and SDS–PAGE. Samples were equilibrated in pH 7.0 (closed state) or pH 4.0 (desensitized state). We used pH 4.0 for DEER measurements due to potential instability issues of membrane scaffolding proteins in nanodisc at more acidic pH conditions. The peak corresponding to pentameric GLIC reconstituted into nanodiscs was collected and flash-frozen in 20% glycerol for DEER measurements.

### DEER measurements

Inter-subunit distances (<50 Å) were measured using Double Electron-Electron Resonance (DEER) methods (Zou and McHaourab, 2010; Jeschke et al., 2002) for spin-labeled samples reconstituted in nanodiscs. Four-pulse DEER experiments were performed using a Bruker ELEXSYS E580 spectrometer equipped with a SuperQ-FT pulse Q-band system with a 10 W amplifier and EN5107D2 resonator. The sample was loaded into a 1.1 mm inner diameter quartz capillary (Wilmad LabGlass, Buena, NJ) and mounted into the sample holder (plastic rod) inserted into the resonator. Dipolar time evolution data were obtained at 80K using a standard DEER four-pulse sequence (π/2)mw1–τ1–(π)mw1–τ1–(π)mw2–τ2–(π)mw1–τ2–echo (Pannier et al., 2000) at Q-band frequency (~33.9 GHz). The experimental conditions were: pulse lengths for (π/2)mw1 and (π)mw1 were 10 and 20 ns, respectively, and 24 ns for (π)mw2, 80 MHz of frequency difference between probe and pump pulse, shot repetition time determined by spin-lattice relaxation rate (T_1_), 100 echo/point, and 2-step phase cycling. Data were collected out to ~2.0 µs for overnight data acquisition time. DEER signals were background-corrected assuming a 3D homogeneous background and analyzed by the Tikhonov regularization in the DEER Analysis 2014 software (Jeschke et al., 2006; Chiang et al., 2005) to determine average distances and distributions in distance. The regularization parameter in the L curve was optimized by examining the fit of the time domain.

### Membrane reconstitution and electrophysiology

Electrophysiological measurements were made by patch clamp recordings in channel-reconstituted liposomes prepared as described earlier (Velisetty and Chakrapani, 2012; Delcour et al., 1989; Cortes et al., 2001; Chakrapani et al., 2007). Purified and spin-labelled GLIC mutants were reconstituted into preformed asolectin vesicles by diluting in 150 mM NaCl, 10 mM MOPS, pH 7.0 (reconstitution buffer). The detergent was removed by incubating the proteoliposome suspension with biobeads overnight at 4°C. The suspension was centrifuged at 100,000g for 1 hr and the pellet re-suspended in reconstitution buffer. A drop of the proteoliposome was placed on a glass slide and dried overnight in a desiccator at 4°C. The sample was then re-hydrated with 20 μl of buffer, which yielded giant liposomes. Channels were reconstituted in 1:10000 protein:lipid (molar ratio) for macroscopic currents. All currents measurements were made at room temperature by inside-out patch-clamping of proteoliposome under symmetrical NaCl concentrations. Recording pipettes were pulled from thin walled borosilicate glass, heat polished to a resistance of 1.5–2 MΩ, and filled with 150 mM NaCl, 10 mM MOPS, pH 7.0. Currents were elicited in response to pH jumps (to pH 3.0, 150 mM NaCl and 10 mM sodium citrate buffer) using an RCS-200 fast solution exchanger (switch time 2 ms) fed by gravity (BioLogic, Knoxville, TN). Currents were measured using Axopatch 200B, digitized at 10 kHz sampling frequency and were analysed using Clampfit 10.2.

### Crystallization

GLIC *wt* in 100 mM NaCl, 10 mM Tris-HCl (pH 7.4), and 0.5 mM DDM was concentrated to between 9–10 mg/ml with an Amicon Ultra 50 KDa cutoff concentrator (EMD Millipore, Billerica, MA). Prior to crystallization setup, the protein was supplemented with 50 μM DHA (from 300 mM DHA stock in ethanol) and 0.5 mg/ml *E.coli* polar extract (Avanti Polar Lipids) and incubated on ice for 1 hr. The protein was crystallized at 4°C by sitting drop vapor diffusion in Cryschem plates (Hampton Research, Aliso Viejo, CA) with a 1:1 mixture (1 μl each) of protein and reservoir solution (225 mM ammonium sulfate, 50 mM sodium acetate, pH 3.9–4.2 and 9–12% PEG4000). Crystals typically formed within one week and typically took 2–3 weeks to reach full size. The crystals were cryoprotected by adding 6 μL reservoir solution supplemented with 30% ethyleneglycol to the drop, and directly frozen in liquid nitrogen using appropriately sized microloops (MiTeGen, Ithaca, NY) or cryoloops (Hampton Research).

### Structure determination

X-ray diffraction data were acquired on NE/CAT beamlines 24ID-C at the Advanced Photon Source at Argonne National Laboratory. The data was indexed using iMosflm (Battye et al., 2011) and further processed using programs within the CCP4 suite(Collaborative Computational Project, 1994). The crystals belong to space group C121 with one pentamer in the asymmetric unit. Initial phases were obtained by molecular replacement using PHASER (McCoy, 2007) with GLIC (PDB ID: 4HFI) (Sauguet et al., 2013) crystal structures as a search model. The initial model was refined with REFMAC5 (Murshudov et al., 1997) and was followed by manual model building/fitting in COOT (Emsley et al., 2010). On each successive cycle of model building and refinement, we analyzed the structure with Molprobity software (Chen et al., 2010). MOLE (Petrek et al., 2007) and HOLE (Smart et al., 1996) software were used to compute pore radius profiles (Absalom et al., 2003).

## Additional files

### Major datasets

The following dataset was generated:

Basak S,Schmandt N,Gicheru Y,Chakrapani S,2016,Crystal Structure of membrane protein.,http://www.rcsb.org/pdb/explore/explore.do?structureId=5J0Z,Publicly available at the RCSB Protein Data Bank (accession no: 5J0Z)

The following previously published dataset was used:

Sauguet L,Corringer PJ,Delarue M,2013,The GLIC pentameric Ligand-Gated Ion Channel at 2.4 A resolution,http://www.rcsb.org/pdb/explore/explore.do?structureId=4hfi,Publicly available at the RCSB Protein Data Bank (accession no: 4HFI)
