# Supplementary material for: Crystal structure and dynamics of a lipid-induced potential desensitized-state of a pentameric ligand-gated channel
Source: eLife. 2017 Mar 6;6:e23886. doi: 10.7554/eLife.23886 (PMC5378477; doi:10.7554/eLife.23886)
Supplement: Related texts

# Article commentary: Decision letter

**DOI:** 10.7554/eLife.23886.032

**Affiliations:** University of Wisconsin-Madison, United States

In the interests of transparency, eLife includes the editorial decision letter and accompanying author responses. A lightly edited version of the letter sent to the authors after peer review is shown, indicating the most substantive concerns; minor comments are not usually included.

Thank you for submitting your article "Crystal Structure and Dynamics of a Lipid-induced Potential Desensitized-State of a Pentameric Ligand-gated Channel" for consideration by *eLife*. Your article has been reviewed by three peer reviewers, one of whom, Baron Chanda (Reviewer #1), is a member of our Board of Reviewing Editors, and the evaluation has been overseen by and Richard Aldrich as the Senior Editor. The following individuals involved in review of your submission have agreed to reveal their identity:Lucia Sivilotti (Reviewer #3).

The reviewers have discussed the reviews with one another and the Reviewing Editor has drafted this decision to help you prepare a revised submission.

Summary:

The prokaryotic ligand gated ion channels like the GLICs and ELICs serve as excellent models to lay the groundwork for structural understanding of mechanisms that underlie gating of pentameric ligand activated ion channels. Despite the fact that there are structures of GABAAR, GlyR and nAChR in a presumably desensitized states there are open questions as to what represents a desensitized conformation (see PMID 20863833). Here, the authors describe the x-ray structure of GLIC, a bacterial pH-gated pentameric ligand gated ion channel, in complex with DHA, a polyunsaturated fatty acid. The structure represents a novel desensitized pore conformation and also provides insight into how DHA, a naturally occurring modulator of nAChR and GABAAR, alters channel function. The structure reveals a heretofore unseen pore conformation, where the lower half of the channel is closed and the upper half is open. To further test whether these conformational changes are observed in a lipid environment, the authors have used EPR and DEER experiments to map conformational changes in transmembrane helices. While the reviewers appreciate the comprehensiveness of this work, they have significant concerns that should be addressed in the revised version.

Essential revisions:

1) The connection between the x-ray structure with the new pore conformation and the EPR experiments studying the M4 helix is confusing to me. All of the referenced studies on the M4 helix being a lipid sensor are from the muscle/torpedo nicotinic receptor. To my knowledge GLIC is not known to sense lipids through M4, and unlike in the nicotinic receptor, mutants in GLIC's M4 helix are well tolerated. Moreover, from what I gather DHA does not interact with M4, and in this new structure, M4 superposes perfectly with that of GLIC in other conformations. Lastly, for all pLGIC for which structures are available in multiple conformations, the M4 helix does not appear to move independently relative to the rest of the helical bundle. The EPR experiments herein are thus hard to reconcile with the current and earlier findings, and might better fit as an independent study. Another reviewer noted that the discrepancy between the structure (very little movement except M2 cf open) and the EPR and DEER data (quite a bit of M4 rearrangement) should be clearly stated. I don't think that we in the field have enough information yet to form a plausible hypothesis to explain the discrepancies of the latter two points. I would not have a particular problem with admitting that we do not understand why these discrepancies occur- these are different proteins in different environments. The work remains interesting and relevant.

2) The GLIC-pH4-DHA structure shows a constriction at I9' position whose radius is 2.5 A. It is not clear why this is considered a gate for desensitized conformation. In the conducting GLIC-pH4, a constriction of similar dimension is seen below at T2' position. In fact, the structure at this position is essentially identical between the conducting and desensitized conformations. It does not appear that this is a steric hindrance for ion flux. Therefore, why is I9' position a desensitized gate. Did the authors make that determination based on lack of resolvable waters and ions below that position?

3) From Figure 3 and its supplementary subsidiaries, it appears that DHA is interacting with GLIC solely via its carboxylate to the arginine guanidinium, and the rest of the lipid is hanging out in space. No contacts are made with the rest of the receptor? Is the Arg118 side chain in a different conformation than in other GLIC structures? Is the beta6-beta7 loop in a different conformation? If there are other contacts and my impression is incorrect, please make a new figure panel or two illustrating in detail the atomic interactions. The conformation of the pore and the mechanism by which lipid stabilizes it stand out to me as the high impact aspects of the study.

4) Patch clamp data from all the mutants in Figure 5—figure supplement 2 all look quantitatively and qualitatively different, which suggests that labeling may have a dramatic effect on function. What are your thoughts on the functionality of these labeled mutants, and is there an estimate of labeling stoichiometry?

5) Related to the above point- The reviewers had concerns as to why the extent of densensitization varies so much. The two electrode voltage clamp is not ideal technique to measure desensitization, as its slow exchange rate means that the peak measurement will be already somewhat distorted. Therefore, the patch clamp data and TEV data are not comparable. Unless not feasible for technical reasons, the authors should stick to just using the patch clamp method with fast exchange for functional characterization. Previous work from the same lab used the much more appropriate technique of fast agonist application to outside-out patches. If you plan to still show both patch clamp and TEV data, please be sure to clarify this in the figure legend to avoid any confusion.

6) In the Introduction, there are several statements that in my opinion should be toned down, as they overemphasize the significance and the robustness of various reports in the literature that do not sound terribly convincing. Desensitization is important, this is a good paper, there is no need to go over the top like this to persuade us that the work is important and had to be done. Introduction paragraph two physiological role of desensitization.… crucial role… I would tone this down. The contribution of desensitisation to synaptic transmission mediated by wild-type receptors is still a subject of discussion. Desensitization is hard to measure. In paragraph two membrane lipids are the key drivers of gating transitions… again please moderate this statement. Paragraph three “Reduced levels [of DHA] are linked with impaired learning ability….” (one of the papers cited is on cardiac channels…). Another reviewer states that it is probably an overstatement to say that a molecular understanding of desensitization is only now beginning to emerge, as there are decades of elegant functional studies and now several structures of eukaryotic receptors that are likely in desensitized states, and moreover, the low pH GLIC structures may represent a desensitized state (see e.g. PMID 20863833).

[Editors' note: further revisions were requested prior to acceptance, as described below.]

Thank you for resubmitting your article "Crystal Structure and Dynamics of a Lipid-induced Potential Desensitized-State of a Pentameric Ligand-gated Channel" for consideration by *eLife*. Your article has been reviewed by two peer reviewers, and the evaluation has been overseen by a Reviewing Editor and Richard Aldrich as the Senior Editor. The following individual involved in review of your submission has agreed to reveal her identity: Lucia Sivilotti (Reviewer #3).

The reviewers have discussed the reviews with one another and the Reviewing Editor has drafted this decision to help you prepare a revised submission.

The revised version looks much improved but the reviewers had lingering concerns which should be addressed.

1) In the first sentence of the Abstract please replace 'crucial' with 'important'.

2). Introduction section, paragraph four should be "a desensitized conformation", not "its desensitized conformation."

3). Please remove the last sentence of the Introduction, or, be more specific about how this new pore conformation is remarkably consistent with decades of research, and provide references.

4) Subsection “Crystal structure of GLIC bound to DHA”, last line of paragraph three, "a" desensitized state, not "the" desensitized state, please.

5) In the same section, paragraph five, please provide a reference for the hydrated radius of Na+.

6) I am copying reviewer's comments verbatim so that you are fully aware of the concerns. These can be addressed by moving some of the speculative parts into the Discussion section.

– Related to the discussion of the desensitization 'gate' paragraph five of subsection “Crystlal structure of GLIC bound to DHA”: there are two underlying assumptions in this paragraph that are supported by little or no experimental evidence. First, the idea that GLIC allows permeation of dehydrated ions, mechanistically akin to what happens in K^+^ channels. Is there experimental evidence (not simulations) that support the notion of GLIC conducting dehydrated ions? Measurements made by Hille and others on eukaryotic members of the family concluded that permeation (in eukaryotes) is mediated by hydrated ions, with open pores having minimum diameters of 6-7.5A (for one example see PMID 6247422). The second assumption is that the low-pH structures of GLIC represent bona fide open states. To be fair we do not really know whether the low pH structures of GLIC represent open, desensitized or some kind of intermediate state. Careful fast-perfusion patch clamp experiments suggest that GLIC should be desensitized at steady state, as per the comment in the first round of review. To my knowledge no one has put a series of inorganic and organic cations through GLIC to define a minimal open channel diameter. In the absence of that kind of data, it seems safest to assume that the GLIC open channel looks roughly like a mammalian receptor open channel- with a constriction of 6-7.5A in diameter, like in the glycine receptor structure, which is even more open than that (from Gouaux's group). This paragraph may fit better in the discussion- up to the authors. Regardless I suggest it is reasonable to in this section discuss the possibility that we are looking rather at a second, perhaps deeper (and lipid-stabilized) desensitized state in this new structure (the authors very nicely touch upon this idea in the discussion). Additionally, there is a large body of structural and functional work now that supports the major desensitization gate being at the base of the channel (classical SCAM experiments, several recent structures, Trevor Smart's GABA-A recent paper on desensitization). GLIC may be different and lets through dehydrated ions, or this observed conformation may only be relevant to GLIC, we do not yet know. I fear that stating strongly that the desensitization gate in GLIC is at the 9' site, and not at the base of the pore, will confuse people, and that the data are not strong enough to support that single interpretation. I do appreciate the point about slowing/decreasing extent of desensitization with polar substitutions at the 9' position. What I would be more comfortable with is modifying this section just a bit to say the conformation of the pore observed in the structure is most likely impermeable to hydrated ions from 9' to the base of the pore, suggesting that the whole lower half of the pore forms the 'gate' in this conformation of GLIC.

7) Reviewers are still concerned that the limitations of TEVC recordings were not acknowledged. We are not asking additional experiments but just that you make it clear that the observed effects on desensitization may be in large part due to shortcomings of the TEVC system for rapid concentration jumps.

– I think I may be misunderstanding something in the author's response to point 5, related to using oocytes and TEVC because little rundown and complete recovery from desensitization are observed. The key point to me about fast perfusion vs. TEVC is that one does not observe the fast desensitization component in TEVC recordings. Thus, one cannot tell in TEVC if there is recovery or not- the observation in the response that recovery is not observed in patches further solidifies the argument that the TEVC results may be misleading. Again, though, I may not be understanding well.

# Reply: Author response

**DOI:** 10.7554/eLife.23886.033

*Essential revisions:*

*1) The connection between the x-ray structure with the new pore conformation and the EPR experiments studying the M4 helix is confusing to me. All of the referenced studies on the M4 helix being a lipid sensor are from the muscle/torpedo nicotinic receptor. To my knowledge GLIC is not known to sense lipids through M4, and unlike in the nicotinic receptor, mutants in GLIC's M4 helix are well tolerated. Moreover, from what I gather DHA does not interact with M4, and in this new structure, M4 superposes perfectly with that of GLIC in other conformations. Lastly, for all pLGIC for which structures are available in multiple conformations, the M4 helix does not appear to move independently relative to the rest of the helical bundle. The EPR experiments herein are thus hard to reconcile with the current and earlier findings, and might better fit as an independent study. Another reviewer noted that the discrepancy between the structure (very little movement except M2 cf open) and the EPR and DEER data (quite a bit of M4 rearrangement) should be clearly stated. I don't think that we in the field have enough information yet to form a plausible hypothesis to explain the discrepancies of the latter two points. I would not have a particular problem with admitting that we do not understand why these discrepancies occur- these are different proteins in different environments. The work remains interesting and relevant.*

We agree with the reviewer in that GLIC is not as sensitive to membrane lipid composition as nAChRs and mutations in M4 are better tolerated. However, from a structural point of view, it is intriguing that the M4 position appears to be fixed with respect to the rest of the TM helices in the pLGIC structures, even though these structures may represent different conformational states. In contrast, functional analysis of M4 mutations in different members of the pLGIC and our EPR data seem to suggest that M4 is dynamic and may undergo a change in environment during gating. Although we cannot explain this disparity with certainty, it is conceivable that M4 movements could be masked in a detergent environment. It is also possible that the lipidic environment around M4 changes without a significant change in the M4 backbone. We believe that high-resolution structural studies of pLGIC in a membrane environment, such as nanodiscs, are needed to address these differences.

*2) The GLIC-pH4-DHA structure shows a constriction at I9' position whose radius is 2.5 A. It is not clear why this is considered a gate for desensitized conformation. In the conducting GLIC-pH4, a constriction of similar dimension is seen below at T2' position. In fact, the structure at this position is essentially identical between the conducting and desensitized conformations. It does not appear that this is a steric hindrance for ion flux. Therefore, why is I9' position a desensitized gate. Did the authors make that determination based on lack of resolvable waters and ions below that position?*

Thank you for raising this point. Given the hydrophobicity of the pore-facing of I9’ sidechains, the permeating ion is likely to be hydrated at this position (radius of hydrated sodium ion: 2.76 Å), and will therefore be occluded in the GLIC- DHA conformation (pore radius 2.4 Å), while it can still permeate through this region in the GLIC-pH4 conformation (pore radius 3.2 Å). On the other hand, although the pore radii at T2’ (GLIC- DHA: 2.5 Å and GLIC-pH4: 2.6 Å) are roughly similar to that of the I9’ position in GLIC-DHA, the T2’ region will not sterically hinder ion permeation since it can coordinate dehydrated sodium ions through T2’ side-chains (radius of dehydrated sodium ion: 1.02 Å). This closure at Ile9’ could also underlie the loss of ion and water occupancy below this position. However, resolution limitation precludes us from making significant interpretations about the conformation at 2’ position.

3) From Figure 3 and its supplementary subsidiaries, it appears that DHA is interacting with GLIC solely via its carboxylate to the arginine guanidinium, and the rest of the lipid is hanging out in space. No contacts are made with the rest of the receptor? Is the Arg118 side chain in a different conformation than in other GLIC structures? Is the beta6-beta7 loop in a different conformation? If there are other contacts and my impression is incorrect, please make a new figure panel or two illustrating in detail the atomic interactions. The conformation of the pore and the mechanism by which lipid stabilizes it stand out to me as the high impact aspects of the study.

As suggested, we have modified the panel in Figure 3—figure supplement 2. Conformational changes in M4 and the β6–β7 loop (including Arg118) are rather small. Besides, a salt-bridge interaction with Arg118, DHA does not appear to engage with the rest of the protein. However it must be noted that the lipid chain is not fully resolved in this structure. We do not exclude the possibility of additional interactions given the remarkable flexibility of the unsaturated fatty acid chain. This is now discussed in the text.

*4) Patch clamp data from all the mutants in Figure 5—figure supplement 2 all look quantitatively and qualitatively different, which suggests that labeling may have a dramatic effect on function. What are your thoughts on the functionality of these labeled mutants, and is there an estimate of labeling stoichiometry?*

Yes, we agree that although spin-labeled M4 mutants exhibit pH dependent activation, the current traces reflect differences in functional properties. These variations may arise from a combination of impacts from cys mutagenesis as well as spin-labeling. The effect on gating kinetics did not come as a surprise considering previous reports in several pLGIC that show large gating changes in M4 mutants. Functional perturbation accompanying SDSL remains a caveat, and we acknowledge this in the text now. Having said that, we also note that since EPR measurements are made at steady-state conditions, the changes in “faster” components of kinetics may not significantly impact the interpretation. We have not determined the labeling stoichiometry for M4 since membrane exposed and peripheral regions of the channel typically have good labeling efficiencies (as was previously determined for the extracellular end of M2 ~90%) (Velisetty, et al. 2012). The signal-to-noise ratio of EPR signals (both in liposomes and nanodisc) were also indicative of good labeling.

*5) Related to the above point- The reviewers had concerns as to why the extent of densensitization varies so much. The two electrode voltage clamp is not ideal technique to measure desensitization, as its slow exchange rate means that the peak measurement will be already somewhat distorted. Therefore, the patch clamp data and TEV data are not comparable. Unless not feasible for technical reasons, the authors should stick to just using the patch clamp method with fast exchange for functional characterization. Previous work from the same lab used the much more appropriate technique of fast agonist application to outside-out patches. If you plan to still show both patch clamp and TEV data, please be sure to clarify this in the figure legend to avoid any confusion.*

It is rightly pointed out that these studies would be much more informative when carried out by patch-clamp electrophysiology using a fast perfusion system. We chose TEVC as the method to study modulation since we were able to observe complete channel recovery during several consecutive DHA pulses with minimal rundown. Patch-clamp measurements from spin-labeled and reconstituted channels were made under conditions that match those of EPR to show that the line shape and environmental parameter changes likely reflect gating conformational changes. We have included this information now in the figure legends.

*6) In the Introduction, there are several statements that in my opinion should be toned down, as they overemphasize the significance and the robustness of various reports in the literature that do not sound terribly convincing. Desensitization is important, this is a good paper, there is no need to go over the top like this to persuade us that the work is important and had to be done. Introduction paragraph two physiological role of desensitization.… crucial role… I would tone this down. The contribution of desensitisation to synaptic transmission mediated by wild-type receptors is still a subject of discussion. Desensitization is hard to measure. In paragraph two membrane lipids are the key drivers of gating transitions… again please moderate this statement. Paragraph three “Reduced levels [of DHA] are linked with impaired learning ability….” (one of the papers cited is on cardiac channels…). Another reviewer states that it is probably an overstatement to say that a molecular understanding of desensitization is only now beginning to emerge, as there are decades of elegant functional studies and now several structures of eukaryotic receptors that are likely in desensitized states, and moreover, the low pH GLIC structures may represent a desensitized state (see e.g. PMID 20863833).*

We have made all the suggested changes in the text.

[Editors' note: further revisions were requested prior to acceptance, as described below.]

*The revised version looks much improved but the reviewers had lingering concerns which should be addressed.*

*1) In the first sentence of the Abstract please replace 'crucial' with 'important'.*

Done

*2). Introduction section, paragraph four should be "a desensitized conformation", not "its desensitized conformation."*

Done

*3). Please remove the last sentence of the Introduction, or, be more specific about how this new pore conformation is remarkably consistent with decades of research, and provide references.*

Done

*4) Subsection “Crystal structure of GLIC bound to DHA”, last line of paragraph three, "a" desensitized state, not "the" desensitized state, please.*

Done

*5) In the same section, paragraph five, please provide a reference for the hydrated radius of Na+.*

Done

*6) I am copying reviewer's comments verbatim so that you are fully aware of the concerns. These can be addressed by moving some of the speculative parts into the Discussion section.*

*– Related to the discussion of the desensitization 'gate' paragraph five of subsection “Crystlal structure of GLIC bound to DHA”: there are two underlying assumptions in this paragraph that are supported by little or no experimental evidence. First, the idea that GLIC allows permeation of dehydrated ions, mechanistically akin to what happens in K^+^ channels. Is there experimental evidence (not simulations) that support the notion of GLIC conducting dehydrated ions? Measurements made by Hille and others on eukaryotic members of the family concluded that permeation (in eukaryotes) is mediated by hydrated ions, with open pores having minimum diameters of 6-7.5A (for one example see PMID 6247422). The second assumption is that the low-pH structures of GLIC represent bona fide open states. To be fair we do not really know whether the low pH structures of GLIC represent open, desensitized or some kind of intermediate state. Careful fast-perfusion patch clamp experiments suggest that GLIC should be desensitized at steady state, as per the comment in the first round of review. To my knowledge no one has put a series of inorganic and organic cations through GLIC to define a minimal open channel diameter. In the absence of that kind of data, it seems safest to assume that the GLIC open channel looks roughly like a mammalian receptor open channel- with a constriction of 6-7.5A in diameter, like in the glycine receptor structure, which is even more open than that (from Gouaux's group). This paragraph may fit better in the discussion- up to the authors. Regardless I suggest it is reasonable to in this section discuss the possibility that we are looking rather at a second, perhaps deeper (and lipid-stabilized) desensitized state in this new structure (the authors very nicely touch upon this idea in the discussion). Additionally, there is a large body of structural and functional work now that supports the major desensitization gate being at the base of the channel (classical SCAM experiments, several recent structures, Trevor Smart's GABA-A recent paper on desensitization). GLIC may be different and lets through dehydrated ions, or this observed conformation may only be relevant to GLIC, we do not yet know. I fear that stating strongly that the desensitization gate in GLIC is at the 9' site, and not at the base of the pore, will confuse people, and that the data are not strong enough to support that single interpretation. I do appreciate the point about slowing/decreasing extent of desensitization with polar substitutions at the 9' position. What I would be more comfortable with is modifying this section just a bit to say the conformation of the pore observed in the structure is most likely impermeable to hydrated ions from 9' to the base of the pore, suggesting that the whole lower half of the pore forms the 'gate' in this conformation of GLIC.*

We agree with the reviewer on this point. As per the suggestion, we have edited this paragraph and moved it to the Discussion section. The Discussion section has been reorganized to accommodate this change.

“The GLIC-pH4-DHA structure presented here reveals a novel lipid-induced conformation in the crystal that is physically distinct from previously observed pLGIC conformations […] Consistent with this idea, reducing the side-chain volume or hydrophobicity at the conserved 9′ position increases the open state stability and decreases the apparent desensitization rate in many members of the pLGIC family.”

*7) Reviewers are still concerned that the limitations of TEVC recordings were not acknowledged. We are not asking additional experiments but just that you make it clear that the observed effects on desensitization may be in large part due to shortcomings of the TEVC system for rapid concentration jumps.*

*– I think I may be misunderstanding something in the author's response to point 5, related to using oocytes and TEVC because little rundown and complete recovery from desensitization are observed. The key point to me about fast perfusion vs. TEVC is that one does not observe the fast desensitization component in TEVC recordings. Thus, one cannot tell in TEVC if there is recovery or not- the observation in the response that recovery is not observed in patches further solidifies the argument that the TEVC results may be misleading. Again, though, I may not be understanding well.*

We now acknowledge the TEVC limitation in the text. “We would like to point out that technical limitations of TEVC, which include slower perfusion rates, preclude us from resolving fast kinetic components of desensitization. We therefore, at this point, cannot ascertain which of the multiple desensitized states that DHA stabilizes.”
